# High-intensity interval and moderate-intensity continuous training on cerebral energy metabolism in older rats

**DOI:** 10.1007/s11357-025-01820-5

**Published:** 2025-07-30

**Authors:** Cécile Marcourt, Claudio Rivera, Jürgen Tuvikene, Antoine Langeard, Eli-Eelika Esvald, Florencia Cabrera-Cabrera, Tõnis Timmusk, Jean-Jacques Temprado, Jérôme Laurin

**Affiliations:** 1 Aix Marseille Univ, INSERM, INMED, Marseille, France; 2 Aix Marseille Univ, CNRS, ISM, Marseille, France; 3 Neuroscience Center, HiLife, University of Helsinki, Helsinki, Finland; 4 Department of Chemistry and Biotechnology, Tallinn University of Technology, Tallinn, Estonia; 5 Normandie Univ, INSERM, COMETE, Caen, France

**Keywords:** Angiogenic markers, Brain health, Lactate threshold, Cortical and hippocampal energy metabolism, Bulk RNA sequencing

## Abstract

Aging is associated with metabolic decline in the brain, increasing susceptibility to neurodegenerative diseases. While exercise is a well-established strategy to counteract these changes, no study has directly compared the effects of moderate-intensity continuous training (MICT) and high-intensity interval training (HIIT) on cortical and hippocampal energy metabolism—key regulators of brain plasticity in aging. To address this gap, we investigated how 4-week MICT and HIIT protocols, structured according to the lactate threshold, affect endurance performance and brain metabolic markers in older Wistar rats. Both training modalities improved endurance, with HIIT demonstrating superior gains in maximal performance. However, molecular analyses revealed that MICT induced more extensive metabolic and angiogenic adaptations in the cortex and hippocampus, including the upregulation of key regulators of energy metabolism and vascularization. RNA sequencing confirmed broader transcriptomic changes following MICT, implicating pathways associated with neurogenesis, metabolic homeostasis, and cellular plasticity. While HIIT provided a time-efficient means of improving cardiovascular endurance and mitochondrial activity through different molecular pathways when compared to MICT, its impact on brain metabolism was more limited. These findings suggest that MICT is the preferred regimen for enhancing cerebral metabolic function and neurovascular adaptation, while HIIT serves as a complementary strategy to involve other brain metabolism-associated pathways and maximize aerobic fitness. A direct comparison of these modalities, as presented here, is essential for refining exercise prescriptions to support brain health in aging.

**Supplementary Information:**

The online version contains supplementary material available at 10.1007/s11357-025-01820-5.

## Introduction

Brain health, broadly defined as the preservation of cognitive functions, gradually declines across the lifespan [1]. This age-related decline is partly driven by changes in the expression of neurotrophic factors, neurogenesis, and synaptic plasticity, primarily affecting the hippocampus and cortex [2–4]. A growing body of evidence suggests that impairments in brain energy metabolism—specifically, the supply and utilization of glucose and oxygen—play a key role in these structural and functional deteriorations. This is supported by findings of reduced expression of key markers involved in glycolysis, glucose transport, and oxidative phosphorylation [1, 5, 6]. Furthermore, the gradual decline in cerebral blood flow further limits the supply of glucose and oxygen to brain cells. This process is closely linked to a decrease in vascular endothelial growth factor (VEGF), a key signaling protein essential for angiogenesis. [6–9]. These metabolic and angiogenic alterations may set the stage for the more severe brain energy deficits observed in Alzheimer’s and Parkinson’s diseases [6, 10], highlighting the urgent need to identify effective strategies to restore brain energy metabolism [11].

Aerobic training has been shown to preserve brain health and reduce the risk of developing neurodegenerative diseases [12, 13]. This phenomenon is partly attributed to the upregulation of brain energy metabolism markers in rodents, which are known to influence neurotrophic processes [14–16]. Among these markers, the AMP-activated protein kinase (AMPKα) is a master signaling protein involved in cellular energy homeostasis. In addition, mitochondrial activity can be reflected by citrate synthase (CS) and the complex 4 of the mitochondrial respiratory chain (COX4), while the peroxisome proliferator-activated receptor γ coactivator 1-alpha (PGC-1α) mirrored mitochondrial biogenesis [12, 13, 17–21]. However, it should be noted that these findings are primarily based on studies using running wheel protocols, which precludes the ability to define the optimal running speed and session duration needed to enhance the brain energy metabolism.

To address this, a comparison of the aerobic exercise regimens, characterized by different running speeds and durations, is necessary. The two main aerobic exercise regimens to be compared are the moderate-intensity continuous training (MICT: high-volume, low-intensity training) and the high-intensity interval training (HIIT: high-intensity, low-volume training). The MICT and HIIT sessions can be individualized from the running speed associated with the lactate threshold (S_LT_), which corresponds to the first rise in blood lactate concentration above resting levels during an incremental exercise test (i.e., the speed at which anaerobic metabolism becomes significantly involved). In these conditions, inter-individual variability is reduced along with a greater efficacy compared to sessions only based on maximal speed (S_max_), defined as the highest running speed corresponding to the last fully completed stage of an incremental treadmill test [22, 23]. Notably, there is a paucity of research directly comparing the effects of standardized (equivalent energy expenditure) and individualized MICT and HIIT on cortical and/or hippocampal energy metabolism, particularly in aging. However, such a comparison may define their molecular signature and contribute to clarifying the exercise guidelines for older adults.

Therefore, the purpose of this study was to determine the relative effects of 4 weeks of either treadmill-based MICT or HIIT on endurance performance and the expression of metabolic and angiogenic markers in the hippocampus and cortex of older rats. To elucidate the cellular and molecular mechanisms underlying the response to MICT and HIIT, cerebral metabolic functions and pathways were also examined using bulk RNA sequencing.

## Material and methods

### Ethics approval statement

All experimental protocols were approved by the Animal Care Committees #14 of Marseille (APAFIS number: #11,558). All animal experiments complied with the ARRIVE guidelines and were carried out in accordance with the U.K. Animals (Scientific Procedures) Act, 1986 and associated guidelines, EU Directive 2010/63/EU for animal experiments. All methods were performed following the relevant guidelines and regulations. The 3Rs principle was applied: (1) Replacement—No substitution in vitro model exists to mimic endurance training effects on hippocampal and cortical markers; (2) Reduction—To reduce the number of animals, the same rats were used for incremental tests, endurance training, and molecular measurements including Western blot analysis and bulk RNA seq; (3) Refinement—Experiments were either non-invasive or without awakening. To preserve animal welfare and reduce stress, rats were housed by 2 per cage. Enriched environment was provided for all animals while avoiding cognitive and physical training. Food and water were given ad libitum, and rats were maintained at 22 °C with a 12-h light/dark cycle. All acquisitions and analyses were performed in a single-blind manner.

### Animals and experimental protocol

Twenty old male Wistar rats (age: 20 ± 2 months; JANVIER, France) were randomly assigned to one of the following groups: (i) MICT (*n* = 7) where rats followed MICT program, (ii) HIIT (*n* = 5), in which rats carried out HIIT program, and (iii) CONTROL (*n* = 8) in which rats did not perform training program. All animals performed an incremental exercise test at 3 points: before training onset (PRE) and after training (POST). At POST, cerebral cortex and hippocampus tissues were harvested 26–30 h after the last exercise test for all subsequent molecular measurements. Before starting protocol, all rats were familiarized with treadmill for 5 days.

Animals were excluded based on pre-established criteria including: (i) inability to perform treadmill running during familiarization or training; (ii) clinical signs of pain or distress (e.g., hunched posture, porphyrin staining, self-isolation); (iii) marked reduction in food/water intake or significant weight loss (> 20%); (iv) development of visible subcutaneous masses impairing locomotion; (v) signs of hypoactivity or unresponsiveness; and (vi) sudden unexplained death unrelated to the intervention. Three animals were excluded during the study: one from the MICT group, one from the HIIT group, and one from the CONTROL group. Exclusion was due to either a suspected subcutaneous mass or sudden death during the protocol. In line with ethical guidelines, decisions were made in consultation with the designated animal welfare officer. No post-mortem examinations were conducted.

### Incremental exercise test on treadmill

It defined the individualized running speed for MICT and HIIT sessions and the gains in endurance fitness. A 1° inclination treadmill was used to perform incremental tests. In order to adjust running speed for rats, a power supply was added [24]. Animals started with a warm-up session at a speed of 6 m min^−1^ for 3 min. Then, for the first step, the running speed was fixed at 8 m min^−1^ and was increased by 2 m min^−1^ every 3 min. The test stopped when the rat could not maintain the imposed running speed. The last speed level reached was considered the S_max_ (m min^−1^). Concurrently, lactate concentration was measured from a small and painless incision at the tail vein following the completion of each speed level to determine the S_LT_. For that purpose, animals were removed from treadmill for a quick period (~ 20 s) to measure lactate concentration (mmol L^−1^) using a portable device (Lactate Scout +, EKF diagnostics, France). The S_LT_ was determined when (1) the inflection point of blood lactate concentration was observed and/or (2) an increase of 1 mmol L^−1^ between two blood lactate values was measured. The incremental test ended when the rat was unable to maintain the imposed running speed **(**Figure S1**)**.

### MICT and HIIT programs

To carefully define training intensity, it has been recommended to use submaximal physiological parameters such as the S_LT_ instead of the VO_2max_, especially in older and pathological conditions [23, 25]. During the week preceding protocols, animals performed familiarization and training sessions with the same experimenter on the same treadmill used for incremental test **(**Figure S1**)**. Before starting protocol, rats started with a 5-day familiarization at low speed (10–15 m min^−1^, 5 min per day). To reduce animal stress, animals were forced to run by simply touching the tail instead of using an electrified treadmill [26].

Animals performed either 4 weeks of MICT or HIIT sessions (5 days per week). Each running session started with a warm-up period for 5 min at low intensity (30% of S_LT_). The HIIT session design was: four series of 4 min of high running speed (80–100% of the variation between S_LT_ and S_max_) separated by active recovery period for 3 min fixed at 30% below S_LT_ for 28 min (including recovery period). Speed remained above S_LT_ and below S_max_ ensuring that HIIT intensity remains higher than S_LT_ to be considered HIIT regimen [27].

For the MICT group, running speed was set at 20% below S_LT_ and was continuous throughout the session. Training protocols was standardized to induce the same energy expenditure [28]. The standardization of training protocols ensures that both MICT and HIIT (isocaloric sessions) induce the same energy expenditure [28]; MICT session duration was conditioned by HIIT workload as follows:

$$Mass\;\left(kg\right)\times Intensity\;\left(m.\;{min}^{-1}\right)\times Duration\;\left(min\right)\times Treadmill\;inclination\;\left(\circ\right)\times9.8\;\left(J/kg.m\right)$$

### Brain molecular measurements

At POST, all animals were anesthetized with intraperitoneal ketamine/xylazine injection (120–20 mg/kg), and both frontal cortex and hippocampus were harvest after the last incremental test at POST (within 26–30 h). One cerebral hemisphere was intended for RNA seq while the other side of the brain was kept for molecular analysis by western blot. Brain tissues were flash-frozen in liquid nitrogen and stored at − 80 °C until analyses. One cerebral hemisphere was intended for RNA seq. Both cortex and hippocampal tissues were lysed in RIPA buffer (150 mM NaCl, 1% Triton X100, 0.1% SDS, 50 mM Tris HCl) which contained protease inhibitors (Complete Mini; Roche). Lysates were then centrifugated at 10,000 g for 30 min at 4 °C. The supernatant was then kept for western blot.

### Western blot analysis

Equivalent amount of proteins (40 μg) was first separated by electrophoresis in Bolt Bis–Tris Plus gel (4–12%; Invitrogen gel, ThermoFischer®) and transferred to nitrocellulose membrane for 7 min for measuring all molecules except for BDNF and CREB analysis. Tris-buffered saline with Tween 20 (TBS-T) containing 5% of bovine serum albumin (BSA) was then used to block membranes for 60 min. Membranes were washed with TBST (3 × 5 min) before being incubated with the primary antibodies on a shaker at 4 °C overnight (Tables S1 and S2). Antibodies were diluted in TBS-T solution containing 2.5% of BSA for all tested proteins except for AMPKα and pAMPKα that were diluted in TBS-T with 5% of BSA. After three washings with TBS-T, membranes were incubated with HorseRadishPeroxidase-conjugated secondary antibodies diluted in TBS-T (5% BSA) on a shaker at room temperature prior to chemiluminescence detection. At least two samples per group were represented on each nitrocellulose membrane to allow comparison between groups (× 2 membranes were used per protein). Membranes were stripped to measure multiple proteins on the same membrane. Membranes were also cut to measure several proteins of different molecular weight and avoid interactions between bands. The signal intensity of each protein of interest was measured according to its molecular weight and normalized to α-tubulin protein levels. Intensity values were expressed relative to CONTROL group. The α-tubulin was also normalized to GAPDH and Ponceau staining to assess the influence of exercise on it. When multiple proteins were tested on the same membrane, they were normalized to the same α-tubulin measurements. Signal intensities were measured with the image analysis software G box (Syngene®). Quantification was performed using Gel Plot Analysis plugin (Fiji®). The full western blot images are shown in Fig. S2 and S3.

For BDNF and CREB measures, 50 μg of total protein and 400 ng of recombinant mature BDNF protein were separated on 15% SDS-PAGE gels and transferred to PVDF membranes. Signal from the anti-mouse IgG-HRP was quenched after measuring BDNF to determine CREB protein levels. After blocking (5% skimmed milk in TBST), membranes were incubated overnight at 4 °C with anti-BDNF antibody followed by 2 h with HorseRadishPeroxidase-conjugated secondary antibody diluted in 2% milk-TBST at room temperature. Immunoblots were developed with SuperSignal™ West Atto Maximum Sensitivity Substrate (Thermo Fisher Scientific) and images captured with the ImageQuant LAS 4000 imaging system (GE HealthCare Life Sciences). To determine CREB protein levels, signal from the anti-mouse IgG-HRP (Thermo Fisher Scientific, A16066, 1:10,000) was quenched with 30% H_2_O_2_ for 20 min at RT. Membranes were then washed with TBST and re-blocked before incubation with anti-CREB antibody overnight at 4 °C in 5% milk-TBST. Incubation with anti-rabbit IgG-HRP (Thermo Scientific, 32,460, 1:5000) was done for 2 h at room temperature in 2% milk, and membranes developed as described above. For equal loading control, membranes were stained with Coomassie solution (0.1% Coomassie brilliant blue R-250 dye, 25% ethanol, and 7% acetic acid). BDNF and CREB signal intensities were normalized to Coomassie staining and to the average of the CONTROL group for each brain region. Signal intensity quantification was performed with the IOCBIO Gel Software [29].

### Bulk RNA sequencing

Total RNA was extracted from the cortex and hippocampus using RNeasy Lipid Tissue kit (Qiagen). Generation of polyA-selected unstranded RNA libraries and 150 bp paired-end sequencing on Novaseq 6000 were performed by Novogene® Company Limited as described before [30]. The raw sequencing reads were quality and adapter trimmed and then mapped to the Rn6 genome (obtained from ENSEMBL) using STAR aligner version 2.7.4a as described before [30]. Differential gene expression analysis was carried out using DeSeq2 v. 1.42.0 (Bioconductor) for cortex and hippocampus separately. The log2 fold changes were shrunk using the ashr adaptive shrinkage estimator from the ashr package. Differential gene expression analysis was carried out using DESeq2 package in R. To interpret the biological meaning of the data, overrepresentation (GO) analysis was performed using Metascape [31]. The upstream regulator analysis was performed using Ingenuity Pathway Analysis (IPA, Qiagen).

### Statistical analysis

Normality and equal standard deviation assumptions were tested using Shapiro–Wilk and Bartlett’s tests, respectively, to use the appropriate statistical test. When the data violated normality or homogeneity of variance assumptions, Welch analysis of variance (ANOVA) or Kruskal–Wallis test were used. One-way ANOVA was followed by Tukey’s post hoc comparison, Welch’s ANOVA was followed by Dunnett’s test, and Kruskal–Wallis comparison was followed by Dunn’s multiple comparison test. Eta-squared (*η*^2^) effect size was also calculated for classical one-way ANOVA as follows: between group sum-of-squares/the total sum-of-squares. Based on the normality and equal standard deviation assumptions, we used repeated measures ANOVA for both S_LT_ and S_max_, and either one-way ANOVA, Welch ANOVA, or Kruskal–Wallis test for western blot analysis. Results were considered significant when *p* < 0.05. All data were analyzed using Prism software and were expressed in mean ± SD for parametric and in median (min to max) for non-parametric tests. To analyze the relationship between metabolic activity markers and endurance performance Pearson’s or Spearman’s correlation coefficient with two-tailed significance was conducted depending on the normality of the data.

## Results

### Both training regimens improved endurance performance, but HIIT was superior

In older adults, higher-intensity exercises improve endurance capacity more than moderate-intensity exercises [32, 33]. To assess whether this holds in older rats, we compared the effects of standardized MICT and HIIT on endurance performance using an incremental exercise test at baseline (PRE) and after a 4-week training (POST) to measure S_LT_ and S_max_ **(**Fig. S1**)**. Repeated measures ANOVA revealed a significant main Time effect for both S_LT_ (*F*_(1,16)_ = 78.92, *p* < 0.0001) and S_max_ (*F*_(1,16)_ = 167.4, *p* < 0.0001). Additionally, a significant Time × Group interaction was observed for S_LT_ (*F*_(2,16)_ = 15.96, *p* < 0.001) and S_max_ (*F*_(2,16)_ = 39.56, *p* < 0.001), suggesting that the magnitude of improvement varied between training groups. Sidak post hoc analysis revealed that within trained groups S_LT_ and S_max_ values were higher in POST than in PRE (*p* < 0.0001). Regarding the differences between groups, Tukey post hoc analysis suggested that S_LT_ and S_max_ values were higher in both MICT (*p* < 0.01) and HIIT (*p* < 0.001) compared to CONTROL in POST (Fig. S1B and C). HIIT group had greater S_max_ compared to MICT in POST (*p* < 0.05) **(**Fig. S1**C)**.

### MICT had greater effect on hippocampal and cortical energy metabolism-related protein expression

Because the efficacy of an endurance exercise program is highly dependent on the dosage between the intensity and the duration, we sought to identify the molecular signature in the cerebral cortex and hippocampus related to energy metabolism specific to HIIT and MICT. We therefore removed these brain regions at 26–30 h after the last exercise test for all molecular analyses.

#### In the cortex

**Fig. 1:**
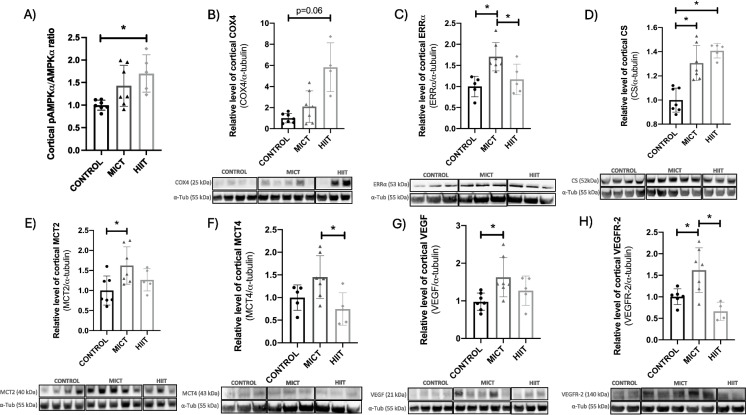
Effect of training regimens on cortical proteins involved in metabolic activity by western blot. Level of cortical **A** pAMPKα/AMPKα ratio (*n* = 19), **B** COX4 (*n* = 18), **C** ERRα (*n* = 17), **D** CS (*n* = 19), **E** MCT2 (*n* = 19), **F** MCT4 (*n* = 17), **G** VEGF (*n* = 19), and **H** VEGFR-2 (*n* = 17) in MICT, HIIT, and CONTROL groups at POST. Pictures of the western blot membranes are shown below each graph. *Significant differences in protein levels between groups. Data is expressed in mean ± SD

First, we measured the levels of AMPKα, as well as COX4, CS, and a key metabolic regulator that interacts with brain-derived neurotrophic factor (BDNF), the estrogen-related receptor *alpha* (ERRα) [16]. Welch ANOVA analysis revealed a significant group effect for COX4 (*F*_(2,5.76)_ = 8.95, *p* < 0.05), and pAMPKα/AMPKα ratio *F*_(2,7.05)_ = 8.48, *p* < 0.05), while one-way ANOVA revealed significant group effect for ERRα (*F*_(2,14)_ = 8.08, *η*^2^ = 0.54, *p* < 0.01) and CS (*F*_(2,16)_ = 22.77, *η*^2^ = 0.74, *p* < 0.0001). Dunnett’s post hoc revealed that HIIT had a higher pAMPKα/AMPKα ratio (*p* < 0.05) and also tended to have higher COX4 levels (*p* = 0.06) relative to CONTROL **(**Fig. 1A, B**).** Tukey post hoc showed higher ERRα in MICT compared to HIIT and CONTROL (*p* < 0.05) while CS was upregulated in both MICT and HIIT compared to CONTROL (*p* < 0.001) **(**Fig. 1C, D**)**. In addition, we investigated the expression of the cerebral lactate transporters following MICT and HIIT. This major energy metabolite is known to promote the expression of neurotrophic factors, including BDNF and VEGF, and may thus mediate cerebral plasticity [21]. One-way ANOVA revealed significant differences between groups for cortical MCT2 (*F*_(2,16)_ = 4.39, *η*^2^ = 0.35, *p* < 0.05) and MCT4 (*F*_(2,14)_ = 4.97, *η*^2^ = 0.42, *p* < 0.05) suggesting that lactate transporters were sensitive to MICT. Indeed, Tukey post hoc demonstrated that MICT had higher MCT2 levels compared to CONTROL (*p* < 0.05), whereas MCT4 expression was lower in HIIT relative to MICT (*p* < 0.05) **(**Fig. 1E, F**)**.

We then examined the influence of endurance training on protein markers of angiogenesis. One-way ANOVA showed significant differences between groups for VEGF (*F*_(2,16)_ = 4.44, *η*^2^ = 0.36, *p* < 0.05) and its receptor VEGFR-2 (*F*_(2,14)_ = 9.58, *η*^2^ = 0.58, *p* < 0.01). Indeed, VEGF levels were higher in MICT compared to CONTROL, while MICT upregulated VEGFR-2 compared to the two other groups (Tukey post hoc: *p* < 0.05 for all) (Fig. 1G, H). No statistical difference between groups was found for AMPKα, pAMPKα, PGC-1α, MFN1, MFN2, MCT1, PECAM-1, the N-methyl-D-aspartate Receptor-1 (GluN1), BDNF, and CREB (Fig. S4 and S6).

#### In the hippocampus

**Fig. 2:**
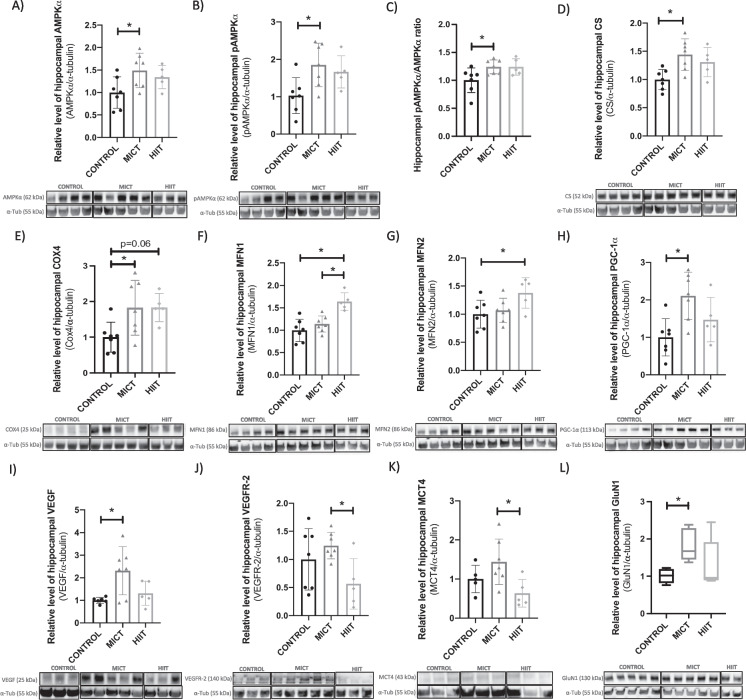
Effect of training regimens on hippocampal proteins involved in metabolic activity by western blot. Level of hippocampal **A** AMPKα (*n* = 19), **B** pAMPKα (*n* = 19), **C** pAMPKα/AMPKα ratio (*n* = 19), **D** CS (*n* = 19), **E** COX4 (*n* = 19), **F** MFN1 (*n* = 19), **G** MFN2 (*n* = 19), **H** PGC-1α (*n* = 19), **I** VEGF (*n* = 18), **J** VEGFR-2 (*n* = 19), **K** MCT4 (*n* = 17), and **L** GluN1 (*n* = 19) in MICT, HIIT, and CONTROL groups at POST. Pictures of the western blot membranes are shown below each graph. *Significant differences in protein levels between groups. Data is expressed in mean ± SD (**A–K**) and in median (min to max) (**L**)

In accordance with studies showing that endurance training improved mitochondrial activity [34], one-way ANOVA demonstrated significant differences between groups for AMPKα (*F*_(2,16)_ = 3.75, *η*^2^ = 0.32, *p* < 0.05), pAMPKα (*F*_(2,16)_ = 4.91, *η*^2^ = 0.38, *p* < 0.05), pAMPKα/AMPKα ratio (*F*_(2,16)_ = 4.32, *η*^2^ = 0.35, *p* < 0.05), COX4 (*F*_(2,16)_ = 4.65, *η*^2^ = 0.38, *p* < 0.05), CS (*F*_(2,16)_ = 6.2, *η*^2^ = 0.44, *p* < 0.05), and for the mitofusin proteins, i.e., MFN1 (*F*_(2,16)_ = 14.13, *η*^2^ = 0.64, *p* < 0.001) and MFN2 (*F*_(2,16)_ = 3.82, *η*^2^ = 0.32, *p* < 0.05). Tukey post hoc showed that MICT upregulated AMPKα, pAMPKα, pAMPKα/AMPKα ratio, CS, and COX4 levels compared to CONTROL (*p* < 0.05), while COX4 also tended to be higher in HIIT compared to CONTROL (*p* = 0.06) **(**Fig. 2A, B, C, D, E**)**. In contrast, while MFN2, protein involved in mitochondrial fusion level, was only higher in HIIT compared to CONTROL (*p* < 0.05), HIIT had higher MFN1 level compared to the two other groups (*p* < 0.01, Tukey post hoc for all) **(**Fig. 2F, G). In the same way, we quantified the master regulator of mitochondrial biogenesis, namely PGC-1α, which is also involved in cerebral plasticity processes by upregulating BDNF. One-way ANOVA revealed significant differences between groups (*F*_(2,16)_ = 6.61, *η*^2^ = 0.45, *p* < 0.01). Higher levels were found in MICT compared to CONTROL (Tukey post hoc: *p* < 0.01) **(**Fig. 2H**)**.

Significant differences between groups were also found for angiogenesis and metabolic activity markers such as VEGF (Welch ANOVA: *F*_(2,6.86)_ = 5.4, *p* < 0.05) and VEGFR-2 (one-way ANOVA: *F*_(2,16)_ = 3.72, *η*^2^ = 0.32, *p* < 0.05). Higher VEGF (Dunnett’s post hoc: *p* < 0.05) were found in the MICT compared to CONTROL (Fig. 2I), while MICT had also higher levels of VEGFR-2 compared to HIIT (Tukey post hoc: *p* < 0.05) (Fig. 2J). Endurance training induced changes in the hippocampal lactate cotransporters MCT4 as shown by significant difference between groups (one-way ANOVA: *F*_(2,14)_ = 4.46, *η*^2^ = 0.39, *p* < 0.05). HIIT downregulated MCT4 expression compared to MICT (Tukey post hoc: *p* < 0.05) (Fig. 2K). Finally, significant differences were found between groups for GluN1, which can be considered as a synaptic plasticity marker (Kruskal Wallis test: (H(2) = 8.979, *p* < 0.01). Dunn’s post hoc suggested that MICT had higher GluN1 level as compared to CONTROL (*p* < 0.01). There was no statistical difference between groups for ERRα, MCT1, MCT2, PECAM-1, CREB, and BDNF (Fig. S5 and S6). No difference was found for α-tubulin normalized by GAPDH and Ponceau staining (Fig. S7).

### MICT induced more extensive transcriptomic changes compared to HIIT

#### Bulk RNA-seq in the cortex

**Fig. 3:**
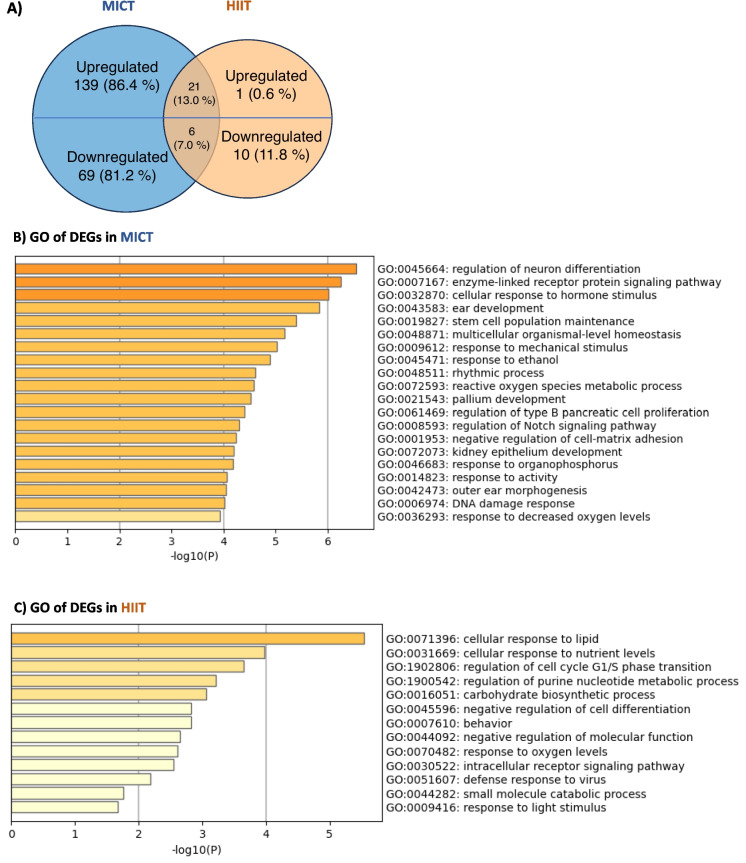
Both training regimens influence metabolic activity and plasticity-associated transcriptional landscape in the cortex. **A** Venn diagram representing the number of differentially expressed genes (DEGs) in both MICT and HIIT groups compared to CONTROL from a bulk RNA sequencing of cortical brain tissue (*n* = 4–8 rats per group). The distribution of genes that were either up- or down-regulated is also shown. Percentages were calculated from the total upregulated and downregulated DEGs (MICT and HIIT). **B** Global DEGs in MICT compared to CONTROL. **C** Global DEGs in HIIT compared to CONTROL

Compared with CONTROL, differentially expressed genes (DEGs) analysis (*p* < 0.05) of MICT and HIIT revealed 235 and 38 DEGs respectively. Of these, 160 and 22 genes were upregulated, while 75 and 16 genes were downregulated in MICT and HIIT groups, respectively. While 27 DEGs were identified as common to both exercise conditions, MICT specifically upregulated 139 genes and downregulated 69 genes, while HIIT only increased the expression of one gene and reduced the expression of ten genes (Fig. 3A).

For example, MICT increased the expression of *Didt4*, *Nfkbia*, *Sgk1*, *Gpd1*, *Olig2*, *Pdk4*, *Nr4a3*, and *Fam107a*, while reducing that of *Ier2* (Fig. S8A). DEGs were associated with various GO terms/pathways including developmental-like processes (e.g., cell differentiation and proliferation), rhythmic process, and metabolic processes (Fig. 3B). Similarly, the genes that were upregulated with MICT were involved in the aforementioned functions (Fig. S8C).

Among others, HIIT strongly increased the expression of *Nfkbia*, *Didt4*, *Sgk1*, *Gpd1*, *Olig2*, and *Tagap* while reducing the expression of *Ier2* and *Tbx1*, involved in different metabolic processes that were observed with MICT (Fig. 3C, S8B, and S8D). While the metabolic process was identified as common to both exercise conditions, MICT influenced specific biological functions also involved in brain development processes (Fig. S11A and B).

To gain further insight into the mechanisms of action of MICT and HIIT, the Ingenuity Pathway Analysis regulator (IPA) was used to predict molecules that may be responsible for changes in gene expression. This tool predicted that MICT activated various upstream regulators including the CREB pathway and the 8 bromo cAMP (which activates the cyclic AMP-dependent protein kinase involved in glucose and lipid metabolism, gene transcription, and cell growth). Furthermore, the IPA predicted the activation of the ERK pathway, which is mediated both by the activation of pathways related to tetradecanoylphorbol acetate and the inhibition of pathways triggered by the ERK/MEK pathway inhibitor PD98059. HIIT was predicted to inhibit fewer upstream regulators activated by MICT, including the dexamethasone phosphate and the dexamethasone (anti-inflammatory effect) dependent pathways (Fig. S9).

#### Bulk RNA-seq in the hippocampus

**Fig. 4:**
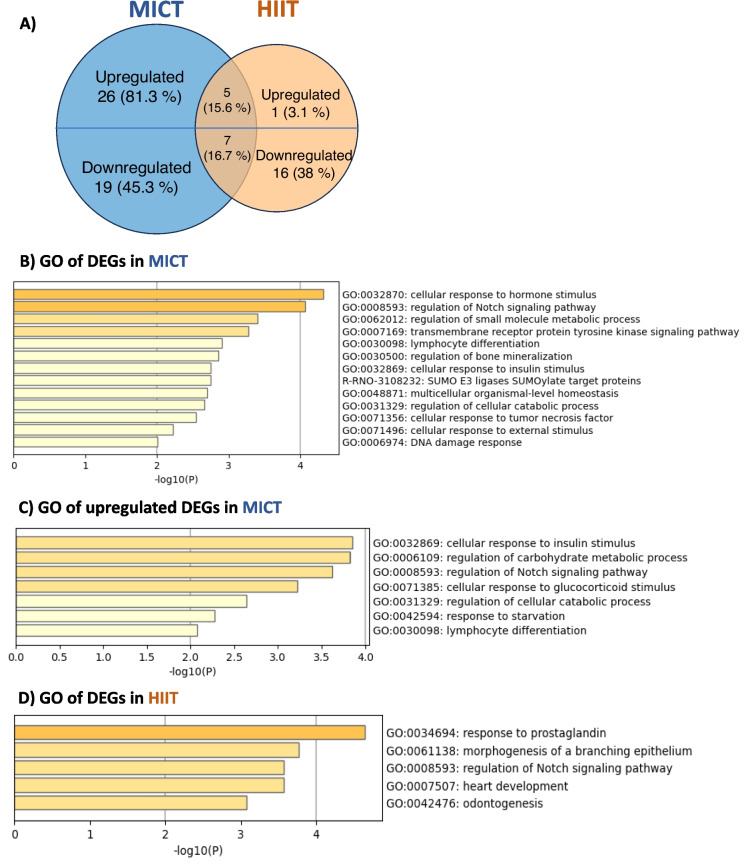
Both training regimens influence metabolic activity and plasticity-associated transcriptional landscape in the hippocampus. **A** Venn diagram representing the number of differentially expressed genes (DEGs) in both MICT and HIIT compared to CONTROL from a bulk RNA sequencing of hippocampal brain tissue (*n* = 4–8 rats per group). The distribution of genes that were either up- or down-regulated is also shown. Percentage was calculated from the total upregulated DEGs (MICT and HIIT) and downregulated DEGs (MICT and HIIT). Volcano plot representing all the genes Gene Ontology (GO) enrichment/pathways analysis of **B** global DEGs and **C** up-regulated DEGs in MICT compared to CONTROL. **D** GO analysis of global DEGs in HIIT compared to CONTROL

Compared with CONTROL, 57 and 29 DEGs were found in MICT and HIIT, respectively (*p* < 0.05). Among them, 31 and 7 genes were upregulated, while 26 and 22 genes were downregulated with MICT and HIIT, respectively. While 27 DEGs were identified as common to both exercise conditions, MICT specifically upregulated 26 genes while downregulating 19 genes. HIIT specifically upregulated only two genes and downregulated 15 genes (Fig. 4A).

MICT mainly increased the expression of *Didt4*, *Mertk*, *Fam107a*, *Btg2*, and *Rbm3* genes, which showed enrichment of GO terms/pathways involved in metabolic and neuronal functions (Fig. 4B and S10A). More specifically, most genes upregulated with MICT were implicated in the regulation of metabolic activity and the Notch signaling pathway (Fig. 4C). The latter is known to play a role in both neurogenic and synaptic plasticity processes.

HIIT mainly increased the expression of *Lfng* and *Tp53bp2* and decreased the expression of *Adamts9*. The corresponding GO terms/pathways included different metabolic pathways than MICT (Fig. 4D and S10B). While the Notch signaling pathway was identified as being common to both exercise conditions, MICT specifically regulated genes expression involved in neuronal energy metabolism and differentiation (Fig. S11C).

The IPA predicted that MICT would stimulate the dexamethasone-activated pathways that have anti-inflammatory effects. For the HIIT, no activation/inhibition prediction was found in the hippocampus (Fig. S10C). The primary networks predicted to be involved in metabolic and neurotrophic activity in the cortex and hippocampus are presented in figs. S12, S13, S14, S15. These figures suggested that MICT promotes stable metabolic adaptations and neurogenesis with reduced stress, while HIIT induces stronger plasticity but requires repair mechanisms, particularly in the hippocampus.

### Endurance performance is correlated with metabolic activity markers

#### In the cortex

Our results showed positive correlation between endurance and some metabolic activity markers. Indeed, S_LT_ was positively correlated with VEGF (*r* = 0.55, large effect size, *p* < 0.05), MCT2 (*r* = 0.61, large effect size, *p* < 0.01), CS (*r* = 0.61, large effect size, *p* < 0.01), and COX4 (*r* = 0.63, large effect size, *p* < 0.01). In the same way, positive correlations were found between S_max_ and MCT2 (*r* = 0.66, large effect size, *p* < 0.01), CS (*r* = 0.61, large effect size, *p* < 0.01), and COX4 (*r* = 0.65, large effect size, *p* < 0.01) (Fig. S16).

#### In the hippocampus

S_LT_ was positively associated with MFN1 (*r* = 0.61, large effect size, *p* < 0.01), MFN2 (*r* = 0.58, large effect size, *p* < 0.05), and COX4 (*r* = 0.66, large effect size, *p* < 0.01). Furthermore, positive correlations were found between S_max_ and MFN1 (*r* = 0.61, large effect size, *p* < 0.01) and COX4 (*r* = 0.65, large effect size, *p* < 0.01) (Fig. S17).

## Discussion

While aerobic exercise is widely used in older adults [11], the question of how different endurance training modalities affect brain energy metabolism remains unanswered. This study demonstrates that MICT strongly promotes genes and proteins related to angiogenesis and energy metabolism in the hippocampus and cortex in accordance with a previous study [35]. However, the potential benefits of HIIT should not be overlooked, as it has been shown to enhance endurance performance in a time-efficient manner and may also exhibit distinct effects on metabolic markers compared to MICT. Given that the rats were untrained prior to the initiation of training protocols, MICT could be prioritized.

Our RNA-seq findings suggested that MICT induced more extensive transcriptomic changes than HIIT in both the hippocampus and cortex. The GO analysis revealed that MICT might improve the regulation of neurogenic and glucose metabolic processes as well as brain cell development processes. MICT also affected rhythmic processes in the cortex whose impairment with age plays a role in health decline by promoting metabolic and inflammatory dysfunction [36]. Our results are consistent with previous findings in older mice reporting that voluntary wheel running reversed a wide range of age-related brain phenotypes (such as neuron projection development) [12]. Furthermore, our findings demonstrated that the HIIT protocol resulted in a reduced activation of pathways associated with lipid and glucose metabolism when compared to the MICT.

Concurrently, at the protein level, our findings demonstrated that MICT and HIIT promoted distinct signaling pathways in both the cortex and hippocampus. This was initially evidenced by the observation that only MICT increased the VEGF pathway, thereby suggesting an enhanced process of angiogenesis in accordance with previous studies conducted in older rodents [37, 38]. VEGF may also play a critical role in neurogenesis and synaptic plasticity [39], which aligns with our findings from RNA-seq analysis. In addition, the IPA prediction tool revealed the activation of ERK after MICT. This pathway has been identified as a key mediator of VEGF expression and is known to be activated by lactate, which is reinforced by the upregulation of the lactate cotransporter MCT2 and MCT4 after MICT in our study [40, 41]. Although IPA predicted the activation of the CREB pathway in the cortex following MICT, our findings showed no change in CREB or BDNF protein levels. This lack of change may be partially attributable to the technique employed for protein revelation, as we recently observed an increase in cortical BDNF concentrations following HIIT, when measured by enzyme-linked immunosorbent assay (ELISA) [42]. Notably, we also observed an increase in expression of its receptors, including the tropomyosin receptor kinase B (TrkB) and (p75 neurotrophic factor or p75^NTR^), following MICT without change in BDNF levels [42]. Specifically, p75^NTR^ is known to potentiate TrkB receptor sensitivity, and thereby enhancing BDNF signaling efficacy even at low ligand levels [43]. This phenomenon has been demonstrated to amplify downstream signaling through pathways such as ERK (in line with the results of the IPA), which has been shown to be critical for synaptic plasticity (as reflected by the upregulation of GluN1 in this study). We underscore that BDNF is not the sole mediator of neuroplasticity after exercise. Other signaling pathways—including those mediated by IGF-1, VEGF, and inflammatory cytokines—contribute to activity-dependent brain remodeling [44]. A potential explanation for the non-significant change in CREB protein expression could be that its pathway activation results in an increase in its phosphorylated form levels rather than CREB. Furthermore, proteins may be subject to post-transcriptional regulation including protein degradation and translational control mechanisms. It is plausible that these mechanisms are responsible for uncoupling mRNA and protein expression for BDNF and CREB. While few studies have examined both exercise-induced mRNA and protein changes in these brain regions, recent research has demonstrated that mRNA increases do not always precede protein changes [45]. Nevertheless, our protein/RNA results converge towards similar conclusions, namely that MICT and HIIT influence metabolic activity and neuronal processes, through different mechanisms of action.

Other findings at the protein level in the hippocampus reinforced that different molecular pathways are involved in MICT and HIIT programs. Indeed, the AMPKα/PGC-1α pathway appeared to be stimulated by MICT. This aerobic metabolism pathway is also implicated in neuroplasticity at gene and protein levels [12, 16, 21, 46]. To reinforce this statement, the hippocampal pAMPKα/AMPKα ratio is increased after MICT reflecting a favorable condition for cerebral plasticity and neuroprotection and could be linked with synaptic plasticity as observed with the upregulation of GluN1 in the hippocampus in our study. In addition, disruption of the AMPKα/PGC-1α pathway has been identified in neurodegenerative diseases such as Alzheimer’s disease, providing potential therapeutic targets [14, 15]. MICT appeared to be a sufficient brain metabolic stressor in older rodents. On the other hand, HIIT increased the levels of mitochondrial fusion proteins MFN1 and MFN2 without altering PGC-1α levels, which is not required to improve energy metabolism [47]. HIIT also increased the pAMPKα/AMPKα ratio but in the cortex and not in the hippocampus as observed only after MICT.

Despite the differences in molecular patterns between MICT and HIIT, these two training protocols shared similar features. Indeed, they both increased cortical CS and hippocampal COX4 protein levels. Moreover, nearly all upregulated cortical DEGs in HIIT were also observed in MICT. The related common GO biological processes in the hippocampus included the positive regulation of the Notch signaling pathway (involved in neurogenic and synaptic plasticity processes) and metabolic processes. Interestingly, positive correlations were observed between endurance performance and several markers of brain metabolic activity, with distinct regional patterns. In the cortex, S_LT_ was positively correlated with VEGF, MCT2, CS, and COX4 levels, while S_max_ was correlated with MCT2, CS, and COX4. These associations highlight the potential link between aerobic performance and both oxidative metabolism and lactate transport capacity, as well as angiogenic activity in cortical tissue. In the hippocampus, S_LT_ showed positive correlations with MFN1, MFN2, and COX4, and S_max_ was associated with MFN1 and COX4. These findings suggest that enhanced endurance capacity may be related to improved mitochondrial dynamics and respiratory chain activity in the hippocampus. Overall, the data point to a region-specific relationship between cardiovascular fitness and brain energy metabolism, emphasizing the involvement of both vascular and mitochondrial adaptations.

Are the preclinical results presented in this study consistent with findings obtained in older human adults? Preclinical studies provide insights into the role of exercise on brain energy metabolism, provided that the exercise can be readily translated to older adults. To this end, our MICT and HIIT sessions were physiologically differentiated using S_LT_ that can also be measured in humans during an incremental exercise test [20, 24]. HIIT had higher speed and MICT longer duration, reflecting human training. Under these conditions, HIIT induced a higher S_max_ in a time-efficient manner than MICT, consistent with a meta-analysis in healthy older adults [48]. In addition, preclinical exercise studies can complement clinical trials by allowing direct measurement of these metabolic markers in specific brain regions, which is not feasible in humans. Since individualized endurance training reduces the risk of injury and optimizes training effectiveness [49, 50], it further supports the translatability of our findings to the human brain.

One limitation of the study is related to the selection of a protein that is known to be unaffected by exercise. To date, all the common proteins of normalization used in western blot analysis might be potentially influenced by exercise. Therefore, we found that our normalization proteins remained stable even after MICT and HIIT to reduce this methodological bias. Another limitation was linked to the small number of animals in RNA sequencing experiments. However, it is a common practice in transcriptomic studies involving aged rodents due to constraints related to availability, cost, and management (mortality rate before the protocol). Importantly, we also used stringent bioinformatics pipelines and statistical criteria (DESeq2, ashr shrinkage) to ensure the robustness of our findings.

## Conclusion

Our findings provide new insights and perspectives on the complementary roles of MICT and HIIT in brain energy metabolism. For practical applications, it is suggested that MICT should be considered as a prioritized exercise regimen for older adults, as it could induce the greatest changes at the transcriptional and protein levels in the hippocampus and cortex, including the involvement of the PGC-1α/AMPKα pathway. However, HIIT should not be excluded from exercise regimens for older adults as it may complement the effects of MICT on cardiovascular fitness and cerebral energy metabolism [51]. In addition, further research is needed to elucidate the mechanisms linking metabolic/angiogenic functions with neurotrophic processes in order to find new therapeutic targets for age-related neurological disorders in accordance with the chemistry of exercise mimetics [52].

## Supplementary Information

Below is the link to the electronic supplementary material.

Supplementary file1 (DOCX 37.2 MB)

## Data Availability

The datasets used and/or analyzed during the current study are available from the corresponding author on reasonable request.
