# Supplementary material for: High-intensity interval and moderate-intensity continuous training on cerebral energy metabolism in older rats
Source: GeroScience. 2025 Jul 30;48(2):2797–812. doi: 10.1007/s11357-025-01820-5 (PMC12972401; doi:10.1007/s11357-025-01820-5)
Supplement: Supplementary file 1 — Supplementary file1 (DOCX 37.2 MB) [file 11357_2025_1820_MOESM1_ESM.docx]

**Supplementary information**

# **High-intensity interval and moderate-intensity continuous training on cerebral energy metabolism in older rats**

Cécile MARCOURT^1,2^; Claudio RIVERA^1,3^; Jürgen TUVIKENE^4^; Antoine LANGEARD^5^; Eli-Eelika ESVALD^4^; Florencia CABRERA-CABRERA^4^; Tõnis TIMMUSK^4^;

Jean-Jacques TEMPRADO^2^; and Jérôme LAURIN^1🖂^

^1^ Aix Marseille Univ, INSERM, INMED, Marseille, France

^2^ Aix Marseille Univ, CNRS, ISM, Marseille, France

^3^ Neuroscience Center, HiLife, University of Helsinki, Helsinki, Finland

^4^ Department of Chemistry and Biotechnology, Tallinn University of Technology, Tallinn, Estonia

^5^ Normandie Univ, UNICAEN, INSERM, COMETE, Caen, France

^🖂^**Corresponding author:**

Dr. Jérôme LAURIN

INMED UMR 1249 – Institut de neurobiologie de la méditerranée

163, avenue de Luminy – BP 13

13273 Marseille cedex 09 - France

**E-mail:** jerome.laurin@univ-amu.fr

**ORCID**: 0000-0001-9692-0060

**Supplementary Figures**

| \| **Antibodies** \| **Species** \| **Company** \| **Dilution** \| **Molecular Weight (kDa)** \| \| --- \| --- \| --- \| --- \| --- \| \| AMPKα \| Rabbit \| Cell Signaling (#2532) \| 1:1000 (BSA 5%) \| 62 \| \| pAMPKα \| Rabbit \| Cell Signaling (#2535S) \| 1:1000 (BSA 5%) \| 62 \| \| VEGF \| Mouse \| Santa Cruz (# Sc-7269) \| 1:200 \| 21-42 \| \| VEGFR-2 \| Mouse \| Sigma-Aldrich (#V3003) \| 1:1000 \| 140 \| \| COX4 \| Mouse \| Santa Cruz (#Sc-69360) \| 1:500 \| 17 \| \| PGC-1α \| Mouse \| Merck (#ST1202) \| 1:1000 \| 38 - 113 \| \| MCT1 \| Mouse \| Santa Cruz (#Sc-365501) \| 1:500 \| 40-48 \| \| MCT2 \| Mouse \| Santa Cruz (#Sc-166925) \| 1:500 \| 40 \|  \| \| MCT4 \| Mouse \| Santa Cruz (# Sc-476140) \| 1:500 \| 43 \| \| PECAM-1 \| Mouse \| Santa Cruz (# Sc-376764) \| 1:500 \| 100 \| \| ERRα \| Mouse \| Santa Cruz (#Sc-65720) \| 1:500 \| 53 \| \| CS \| Mouse \| Santa Cruz (#Sc-390693) \| 1:1000 \| 52 \| \| MFN1 \| Mouse \| Santa Cruz (#sc-166644) \| 1:1000 \| 86 \| \| MFN2 \| Mouse \| Santa Cruz (sc-100560) \| 1:1000 \| 86 \| \| BDNF \| Mouse \| Icosagen (#327–100), clone 3C11 \| 1:1000 \| 14 \| \| CREB \| Rabbit \| Cell Signaling, (#4820) D76D11 \| 1:2500 \| 43 \| \| GluN1 \| Mouse \| Thermofisher (#32-0500) \| 1:1000 \| 110 \| \| α-Tubulin \| Mouse \| Invitrogen (#62204) \| 1:10 000 \| 55 \| \|  \|  \|  \|  \|  \| |
| --- | --- | --- | --- | --- | --- | --- | --- | --- | --- | --- | --- | --- | --- | --- | --- | --- | --- | --- | --- | --- | --- | --- | --- | --- | --- | --- | --- | --- | --- | --- | --- | --- | --- | --- | --- | --- | --- | --- | --- | --- | --- | --- | --- | --- | --- | --- | --- | --- | --- | --- | --- | --- | --- | --- | --- | --- | --- | --- | --- | --- | --- | --- | --- | --- | --- | --- | --- | --- | --- | --- | --- | --- | --- | --- | --- | --- | --- | --- | --- | --- | --- | --- | --- | --- | --- | --- | --- | --- | --- | --- | --- | --- | --- | --- | --- | --- | --- | --- | --- | --- | --- |
| **Table 1: Primary antibodies used for both cortex and hippocampal analysis by Western blot.** Membranes were either incubated with the AMP-activated protein kinase α (AMPKα) and its phosphorylated form (pAMPKα), the Vascular Endothelial Growth Factor (VEGF) and its receptor (VEGFR-2), the Cytochrome c oxidase subunit 4 (COX4), the Peroxisome proliferator-activated receptor-Gamma coactivator (PGC-1α), the Monocarboxylic Acid Transporters 1 (MCT1), 2 (MCT2), 4 (MCT4), the CD-31-PECAM-1 (Platelet endothelial cell adhesion molecule), the ERRα (estrogen-related receptor alpha), the CS (Citrate Synthase), the Mitofusin 1 (MFN1) and 2 (MFN2), the Brain Derived Neurotrophic Factor (BDNF), the CREB (cAMP Response Element-Binding Protein) and the N-methyl D-aspartate Receptor Subunit 1 (GluN1). The α-Tubulin was used for the normalization of proteins. |

| \| Secondary Antibodies \| Species \| Company \| Dilution \| \| --- \| --- \| --- \| --- \| \| Anti-mouse IgG \| Goat \| Thermo Fisher Scientific (#21040) \| 1:5000 \| \| Anti-rabbit IgG \| Goat \| Thermo Fisher Scientific (#21234) \| 1:5000 \| |
| --- | --- | --- | --- | --- | --- | --- | --- | --- | --- | --- | --- | --- |
| **Table 2: Secondary antibodies used for both cortex and hippocampal analysis by Western Blot.** Depending on the species of the primary antibody, membranes were either incubated with anti-mouse or anti-rabbit secondary antibodies. |

|  |
| --- |
| **Figure S1: Experimental design and effect of training regimens on endurance capacity. A)** All trained animals carried out 4 weeks of training, 5 times per week either in MICT or HIIT. All animals performed incremental exercise test in PRE and POST but also at 2 weeks of training to recalculate exercise intensity by considering animals’ progression throughout the protocol. During the incremental test, blood lactate was collected at every level to determine the S_LT_. At the end of the protocol (26-30 hours after the last incremental test) both hippocampi and cortices were removed to perform Western blot measurements and bulk RNA sequencing analysis. Results of **B)** S_LT_ and **C)** S_max_ obtained in PRE and POST in MICT, HIIT and CONTROL (n=5-7 rats per group). *Higher S_LT_ and S_max_ in MICT (p<0.01) and HIIT (p<0.001) as compared to CONTROL in POST. ^+^Higher S_LT_ and S_max_ in MICT and HIIT in POST as compared to PRE (p<0.0001 for all comparison). ^@^Higher S_max_ in the HIIT group as compared to the MICT group in POST (p<0.05). S_LT_: Speed associated with the lactate threshold; S_max_: Maximal speed. |

| **a)**  **** |  |
| --- | --- |
| **b)**  **** |  |
| **c)** |  |
| **d)**  **** |  |
| ****  **e)** |  |
| **f)** |  |
| **g)** |  |
| **Figure S2: Original Western blot membrane images for significant cortical proteins.** There are 2 membranes for each protein: (**a**) COX4, (**b**) ERR-α, (**c**) CS (**d)** MCT2, (**e**) MCT4, (**f**) VEGF and (**g**) VEGFR-2. Groups were separated by dashed lines. The samples derived from the same experiment and that gels/blots were processed in parallel the same day (2 gels per protein to reach a sufficient number of samples). We cut the membranes before exposure to be able to measure proteins in the same membrane for proteins with different molecular weight. |  |
| \| **a)** \| \| --- \| \| **b)** \| \| **c)**  **** \| \| **d)** \| \| **e)** \| \| **f)** \| \| **g)** \| \| **h)** \| \| 1. **** \| \| **j)** \| \| **k)** \| \| **Figure S3: Original Western blot membrane images for significant hippocampal proteins.** There are 2 membranes for each protein: (**a**) AMPKα, (**b**) pAMPKα, (**c**) CS, (**d**) COX4, (**e**) MFN1, (**f**) MFN2, (**g**) PGC-1α, (**h**) VEGF, (**i**) VEGF-R2, (**j**) MCT4 and (**k**) GluN1. Groups were separated by dashed lines. The samples derived from the same experiment and that gels/blots were processed in parallel the same day (2 gels per protein to reach a sufficient number of samples). We cut the membranes before exposure to be able to measure proteins in the same membrane for proteins with different molecular weight. \| |  |
| \|  \| \| --- \| \| **Figure S4: No significant effect of MICT and HIIT on cortical proteins by Western blot.** Results of cortical **(A)** AMPK-α, **(B)** pAMPKα**, (C)** PGC-1α, **(D)** MFN1**, (E)** MFN2, **(F)** MCT1, **(G)** PECAM-1 and **(H)** GluN1 levels in MICT, HIIT and CONTROL groups. No significant difference (n.s) was observed between groups. We cut the membranes before exposure to be able to measure proteins in the same membrane for proteins with different molecular weight (n=5-7). \| | |
| \|  \| \| --- \| \| **Figure S5: No significant effect of MICT and HIIT on hippocampal proteins by Western blot.** Results of hippocampal **(A)** ERRα, **(B)** MCT1, **(C)** MCT2 and **(D)** PECAM-1 levels in MICT, HIIT and CONTROL groups. No significant difference (n.s) was observed between groups. We cut the membranes before exposure to be able to measure proteins in the same membrane for proteins with different molecular weight (n=5-7). \|   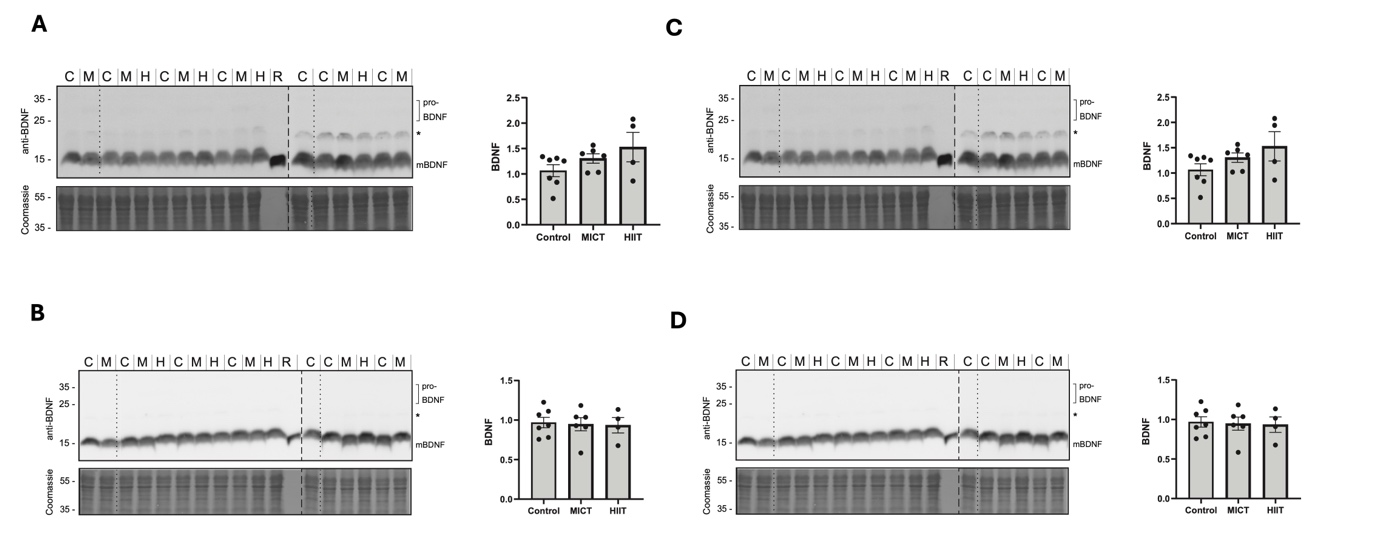 | |
| **Figure S6: No significant effect of MICT and HIIT on cortical and hippocampal BDNF and CREB protein expression.** BDNF protein levels were determined in cortex (A) and hippocampus of aged rats that had undergone Moderate Intensity Continuous Training (MICT, M) or High Intensity Interval Training (HIIT, H) as well as age-matched controls (CONTROL, C) by Western Blot. Recombinant mature BDNF (mBDNF) was included as a positive control. Note that proBDNF is not detected in these samples. The asterisk indicates an unspecific signal. Individual blots are separated by a dashed line. CREB protein levels were also measured by western blot on the same animals in both the cortex (B) and the hippocampus (D). Quantification of both BDNF and CREB signal intensity normalized to Coomassie staining intensity and presented in relation to the average of the control group is shown to the left of the corresponding blots. Each point represents a biological replicate (n=4-7). | |

| **** |
| --- |
| **Figure S7: No significant effect of MICT and HIIT on relative level of α-tubulin by Western blot.** Results of cortical α-tubulin normalized by the GAPDH protein **(A)** and the ponceau staining respectively **(B)** in MICT, HIIT and CONTROL groups**.** Results of hippocampal α-tubulin normalized by the GAPDH protein **(C)** and the ponceau staining respectively **(D)** in MICT, HIIT and CONTROL groups. No significant difference (n.s) was observed between groups. We cut the membranes before exposure to be able to measure proteins in the same membrane for proteins with different molecular weight (n=5-7). |
|  |
| **Figure S8: Both training regimens influence metabolic activity and plasticity-associated transcriptional landscape in the cortex. A**) Volcano plot representing all the genes from the comparison of MICT and CONTROL and **B**) from HIIT and CONTROL. All the DEGs are represented in dark blue and orange (corresponding to a p-value<0.05) and the 10 most significant DEGs are identified in light blue and orange for MICT and HIIT, respectively. Gene Ontology (GO) enrichment/pathways analysis of GO analysis of **C)** up-regulated DEGs in MICT and **D)** up-regulated DEGs in HIIT compared to CONTROL. |

| 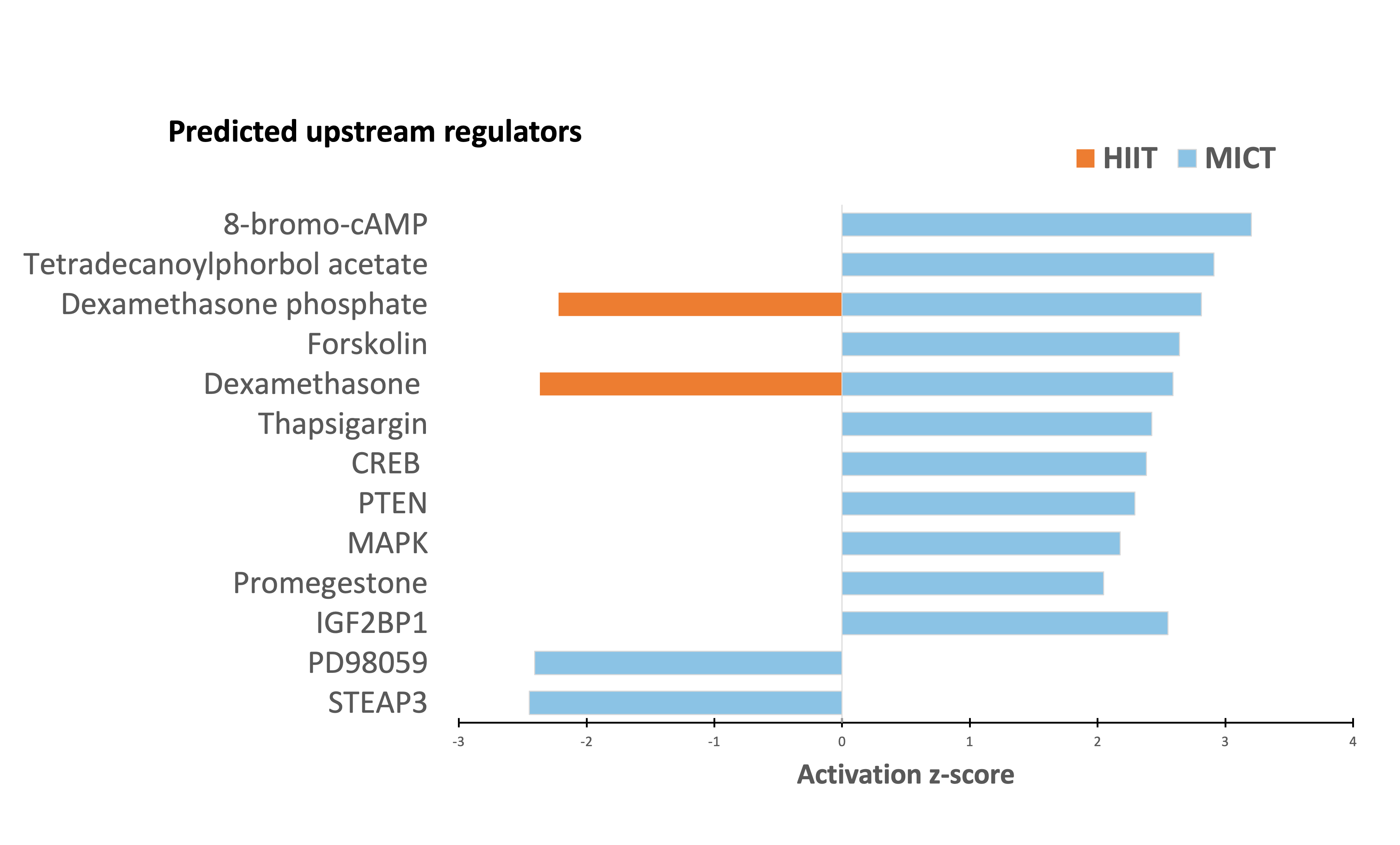 |
| --- |
| **Figure S9: Both training regimens influence metabolic activity and plasticity-associated transcriptional landscape in the cortex.**  Top upstream regulators identified by Ingenuity Pathway Analysis (IPA) for all DEGs in MICT (blue) and HIIT (orange) compared to CONTROL. |
|  |
| **Figure S10: Both training regimens influence metabolic activity and plasticity-associated transcriptional landscape in the hippocampus.** Volcano plot representing all the genes **A**) from the comparison of MICT and CONTROL and **B**) from HIIT and CONTROL. **C)** Top upstream regulators identified by IPA for all DEGs in MICT (blue) compared to CONTROL. |

| A)  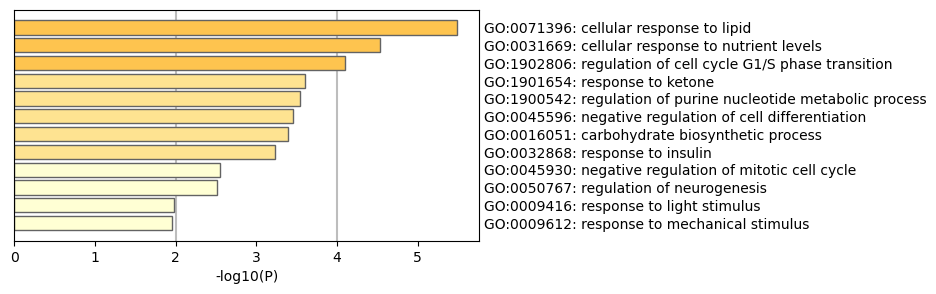 |
| --- |
| B)  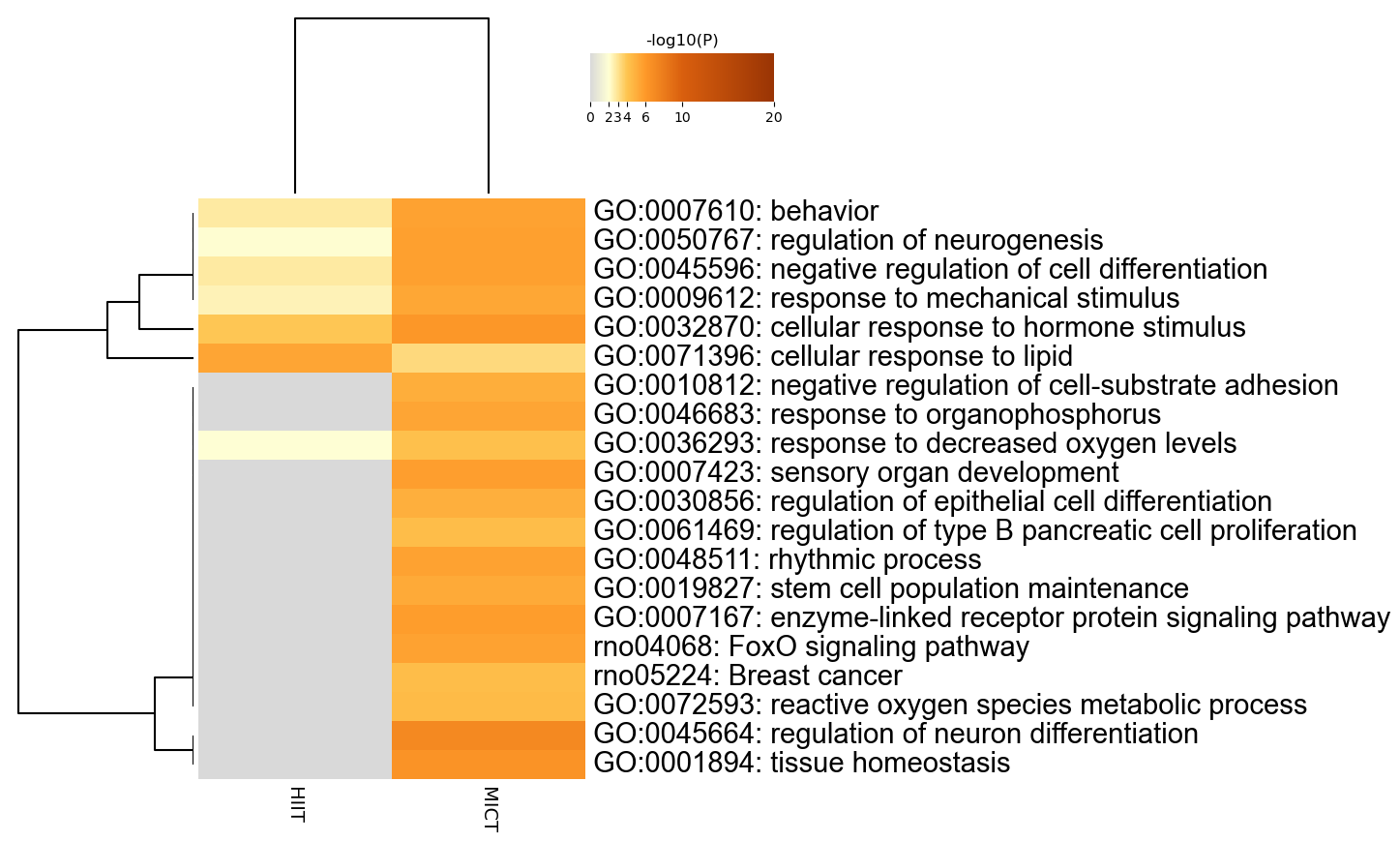 |
| C)  **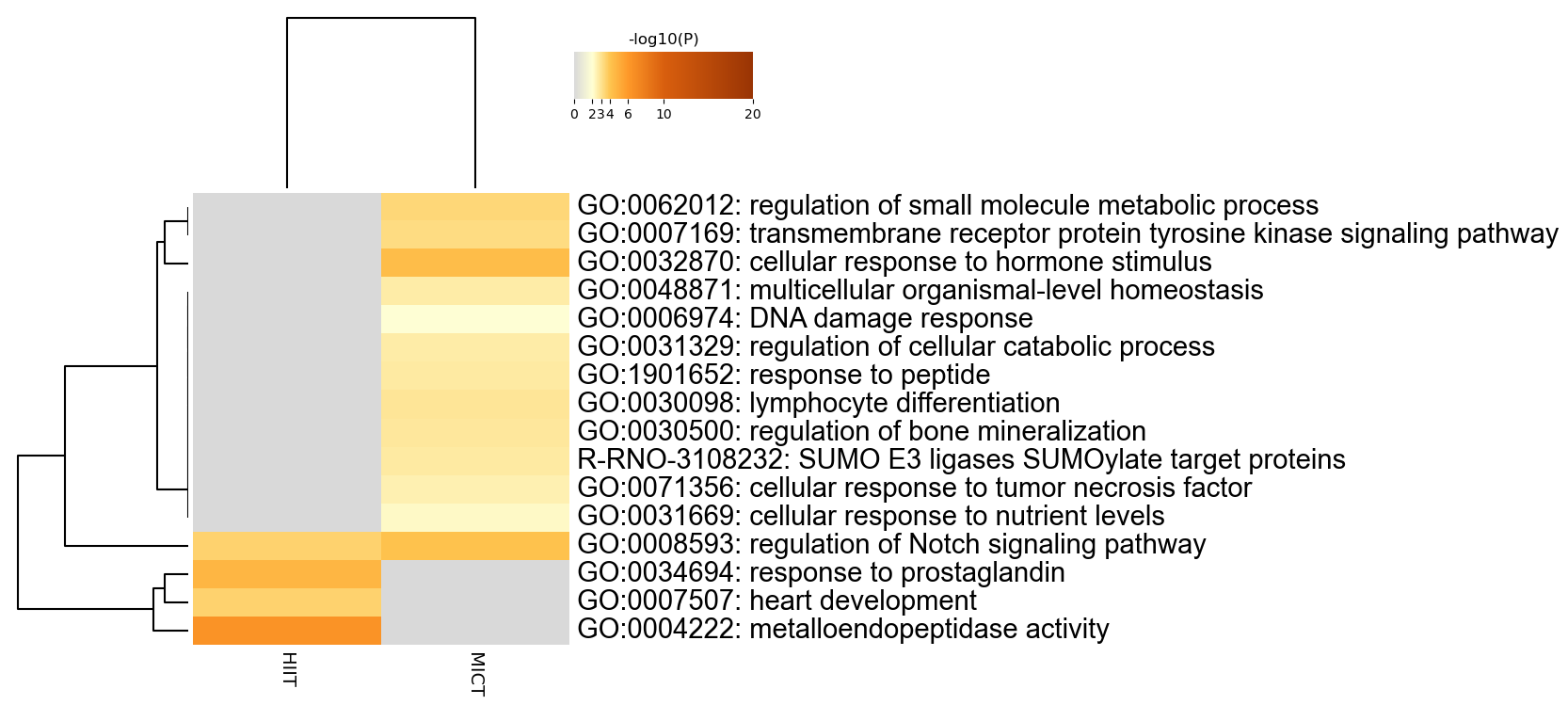** |
| **Figure S11: Common DEGs between HIIT and MICT.** **A)** The specific GO enrichment/pathways analysis of the 21 common DEGs between MICT and HIIT compared to CONTROL in the cortex. **Heatmap between MICT and HIIT. B)** in the cortex, **C)** in the hippocampus. |

| **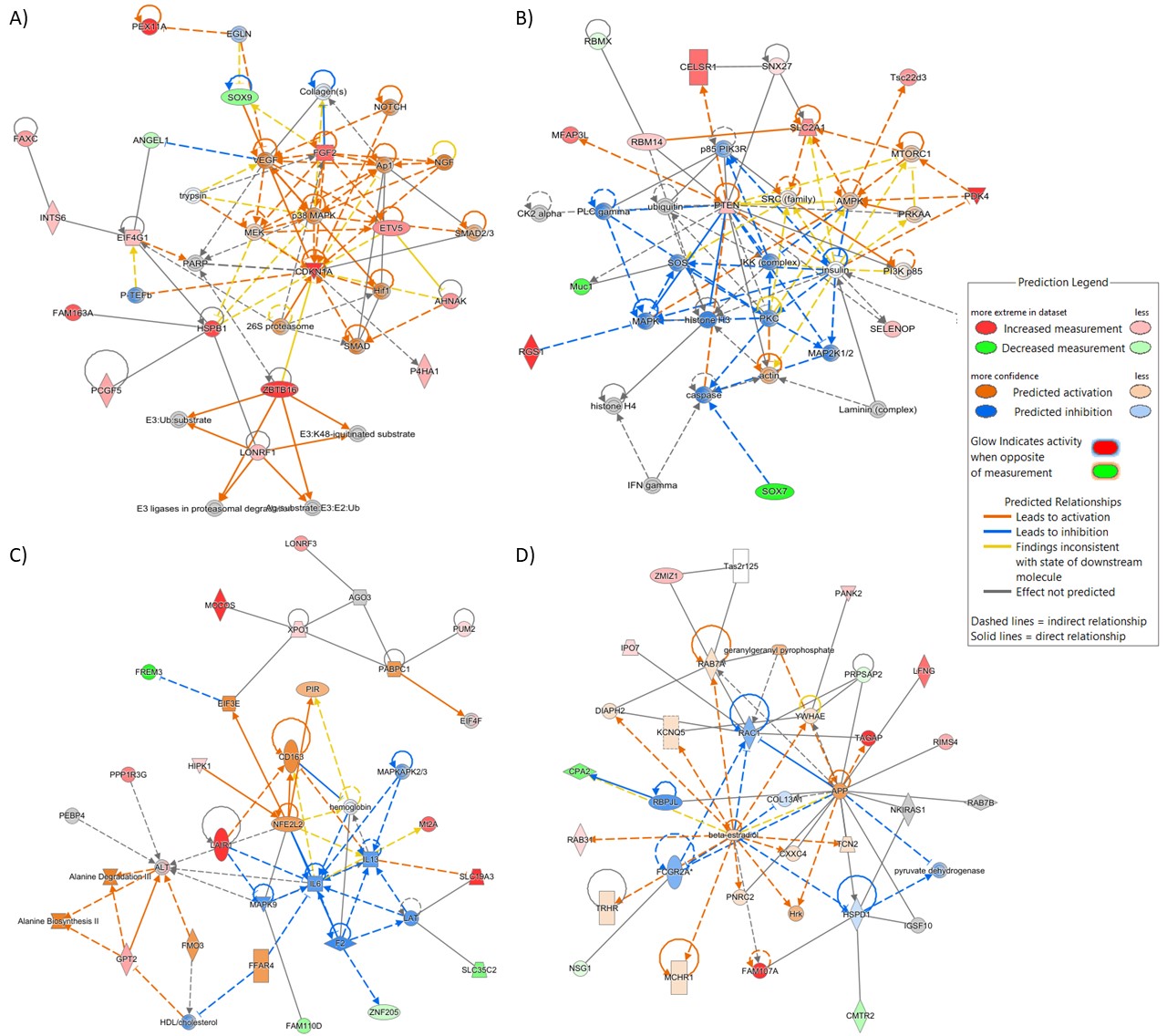** |
| --- |
| **Figure S12: The main predicted networks involved in the MICT condition in the cortex.** The IPA tools revealed that MICT influences different networks in the cortex including the:  A) cell cycle and morphology, cellular growth and proliferation,  B) the carbohydrate metabolism, molecular transport and small molecules biochemistry,  C) lipid metabolism, molecular transport and small molecule biochemistry  D) cell morphology and cellular assembly, organization and development.  Therefore, the activation of cell cycle and proliferation pathways suggests structural adaptation in the cortex, potentially linked to neuronal plasticity and glial cell turnover. Carbohydrate and lipid metabolism are essential for brain energy supply, indicating an optimization of energy substrates in response to training. The impact on cell morphology and tissue organization may reflect changes in neuronal and synaptic architecture, promoting efficient information processing. |

| **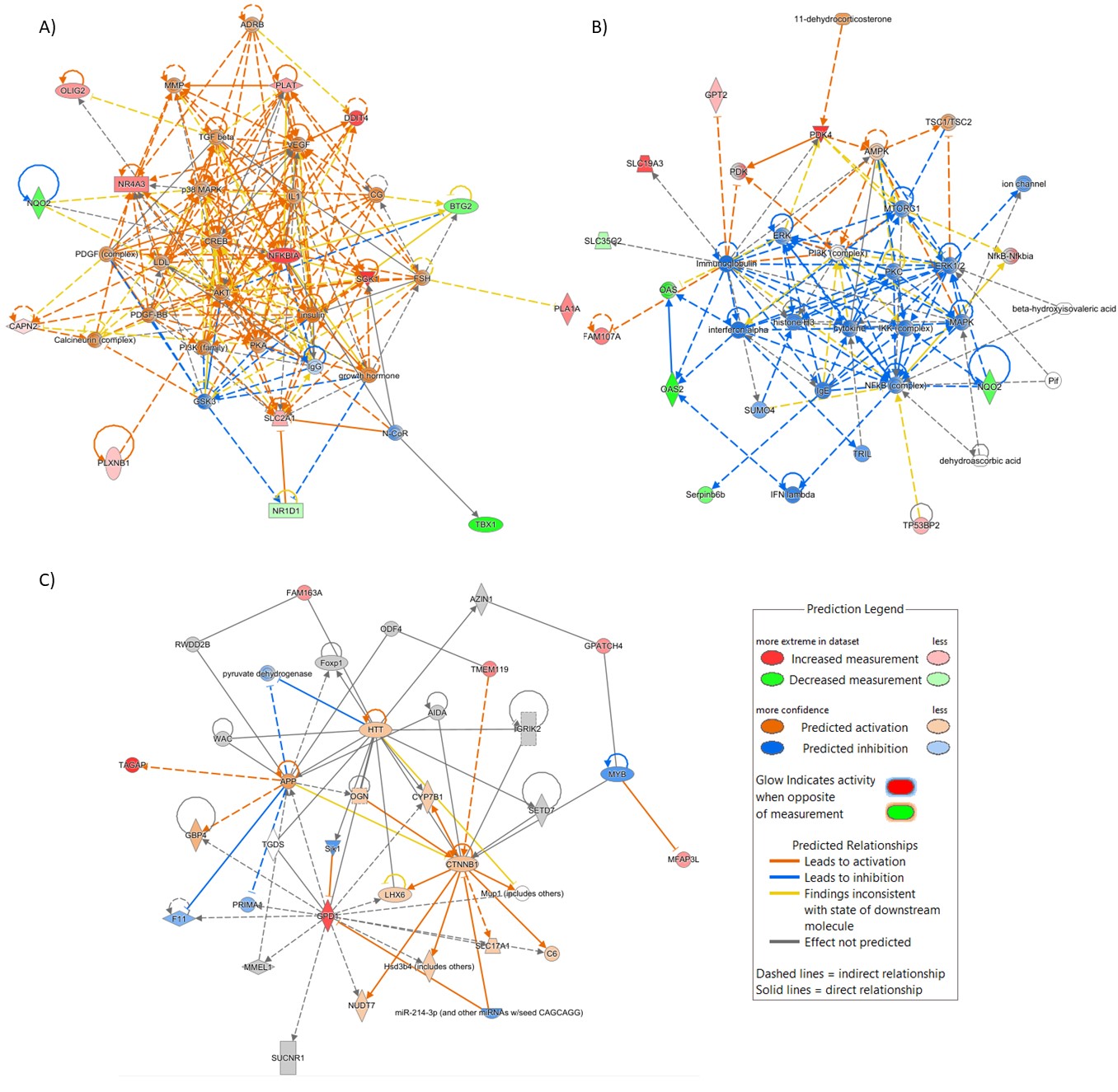** |
| --- |
| **Figure S13: The main predicted networks involved in the HIIT condition in the cortex.** The IPA tools revealed that HIIT influences different networks in the cortex including the:  A) cellular death and survival, cellular movement, organismal injuries and abnormalities,  B) carbohydrate metabolism, small molecules biochemistry, vitamin and mineral metabolism and  C) cell death and survival, cell morphology, and nervous system development.  Therefore, the involvement of cell death and survival pathways suggests that HIIT imposes greater stress on the cortex, triggering adaptive responses involving repair and neuronal remodeling mechanisms. Changes in vitamin and mineral metabolism may reflect an increased regulation of enzymatic cofactors involved in synaptic plasticity and neurotransmission. Effects on nervous system development suggest changes in the formation of new neuronal connections or strengthening of existing networks. |
| **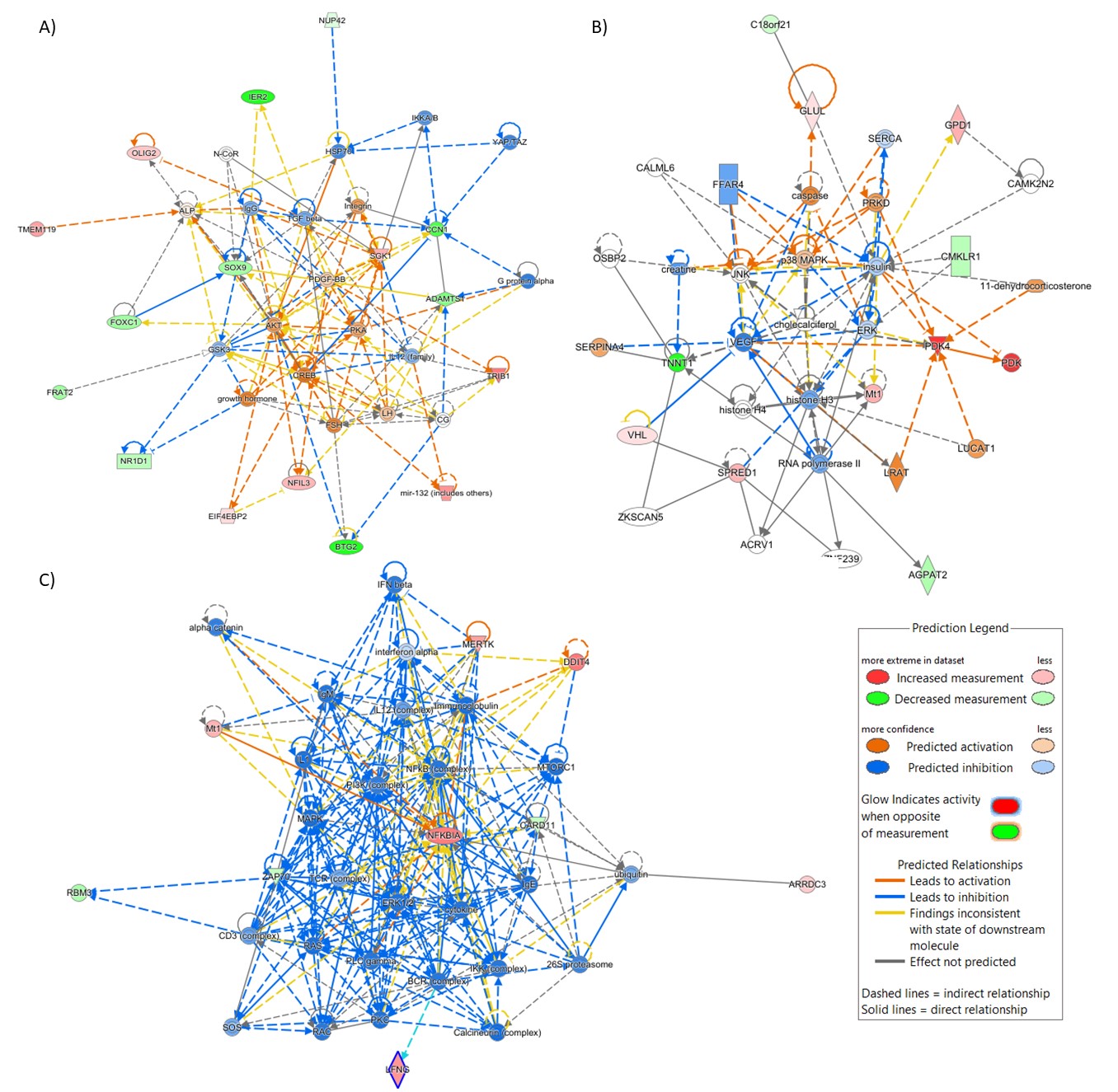** |
| **Figure S14: The main predicted networks involved in the MICT condition in the hippocampus.** The IPA tools revealed that MICT influences different networks in the hippocampus including the:  A) cellular development, growth and proliferation, the embryonic development,  B) lipid metabolism, molecular transport, small molecule biochemistry and  C) cell mediated immune response, cellular development, function and maintenance.  Therefore, the activation of cell development and proliferation pathways is a key marker of hippocampal neurogenesis, essential for learning and memory. The involvement of lipid metabolism suggests adjustments in energy utilization and neuronal membrane synthesis, which are crucial for synaptic plasticity. The modulation of immune response may indicate an anti-inflammatory effect, beneficial for cerebral plasticity. |
|  |

| **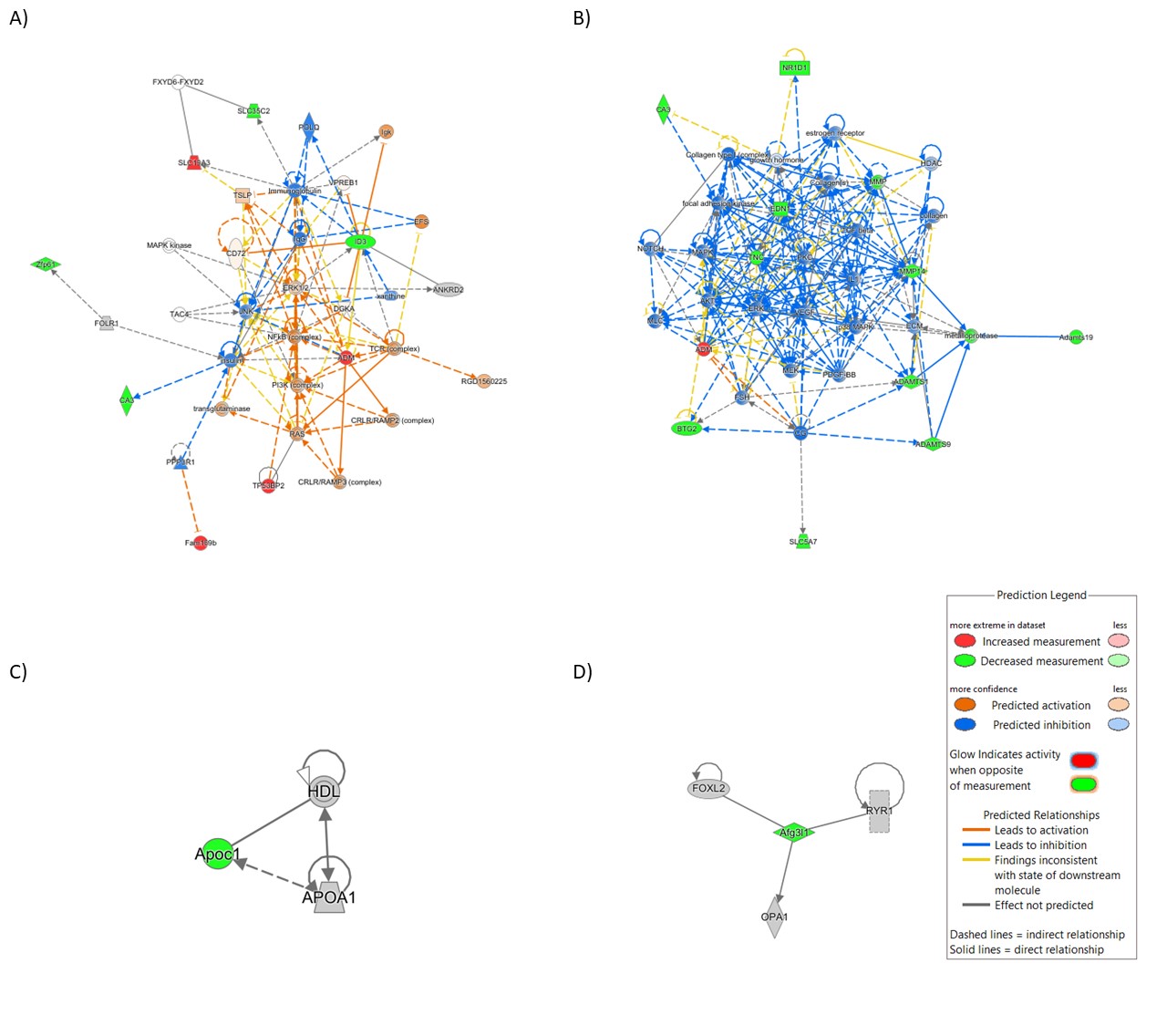Figure S15: The main predicted networks involved in the HIIT condition in the hippocampus.** The IPA tools revealed that HIIT influences different networks in the hippocampus including the:  A) **c**ellular development, growth and proliferation, hematological system development and function,  B) cardiovascular system development and function, cellular movement and endocrine system disorders,  C) carbohydrate metabolism, cellular function and maintenance, protein synthesis  D) cellular assembly and organization, cellular function and maintenance, DNA replication, recombination and repair.  Therefore, the effect on cell development and proliferation confirms that HIIT stimulates neurogenesis, possibly more than MICT. The involvement of the cardiovascular system may suggest an improvement in cerebral blood flow, increasing oxygen and nutrient supply. The activation of DNA repair pathways indicates a response to cellular stress, requiring increased maintenance mechanisms. |
| --- |

| A)  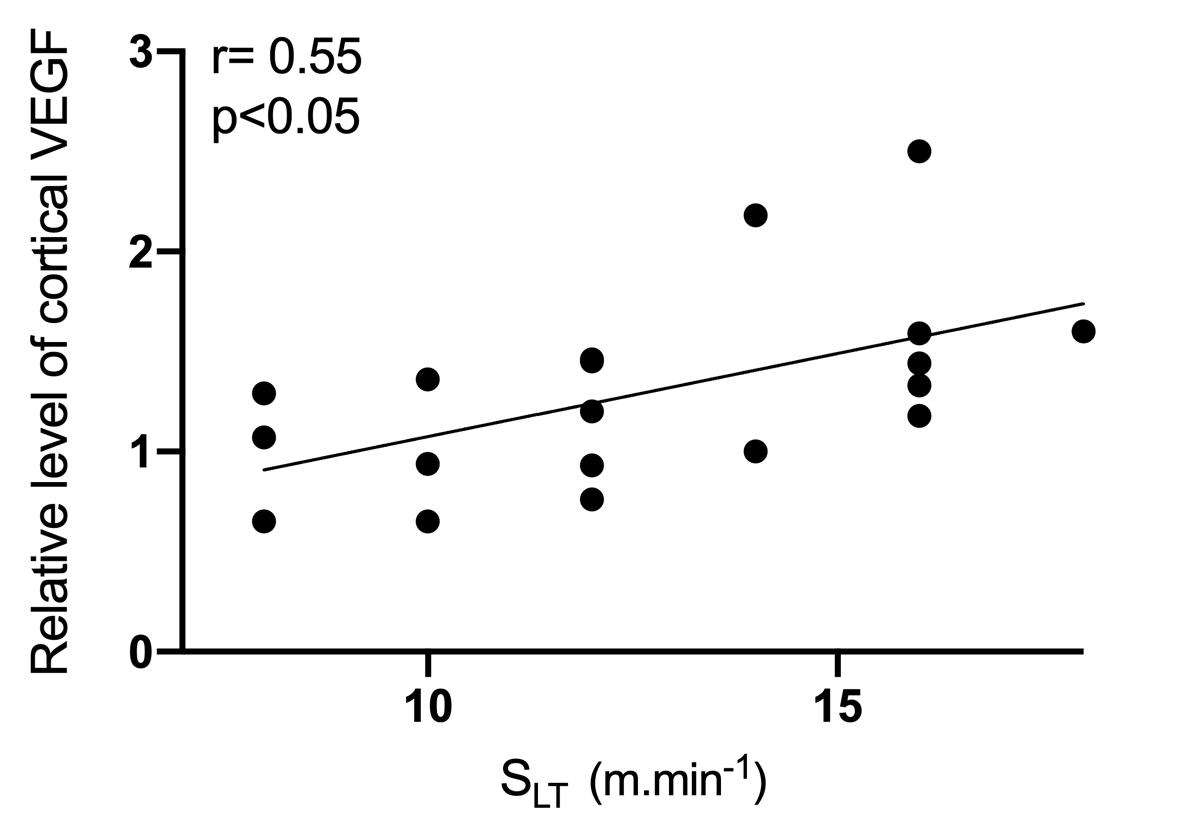 | B)  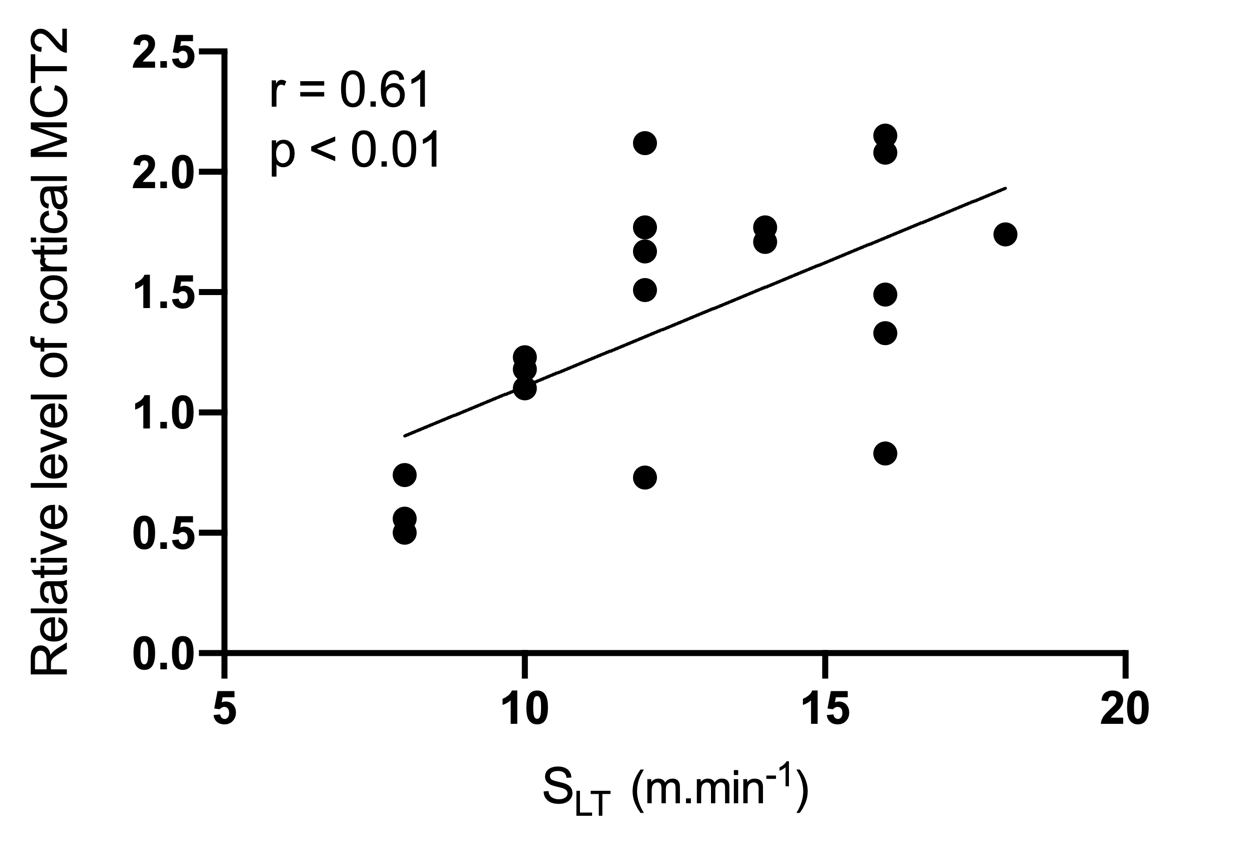 |
| --- | --- |
| C) 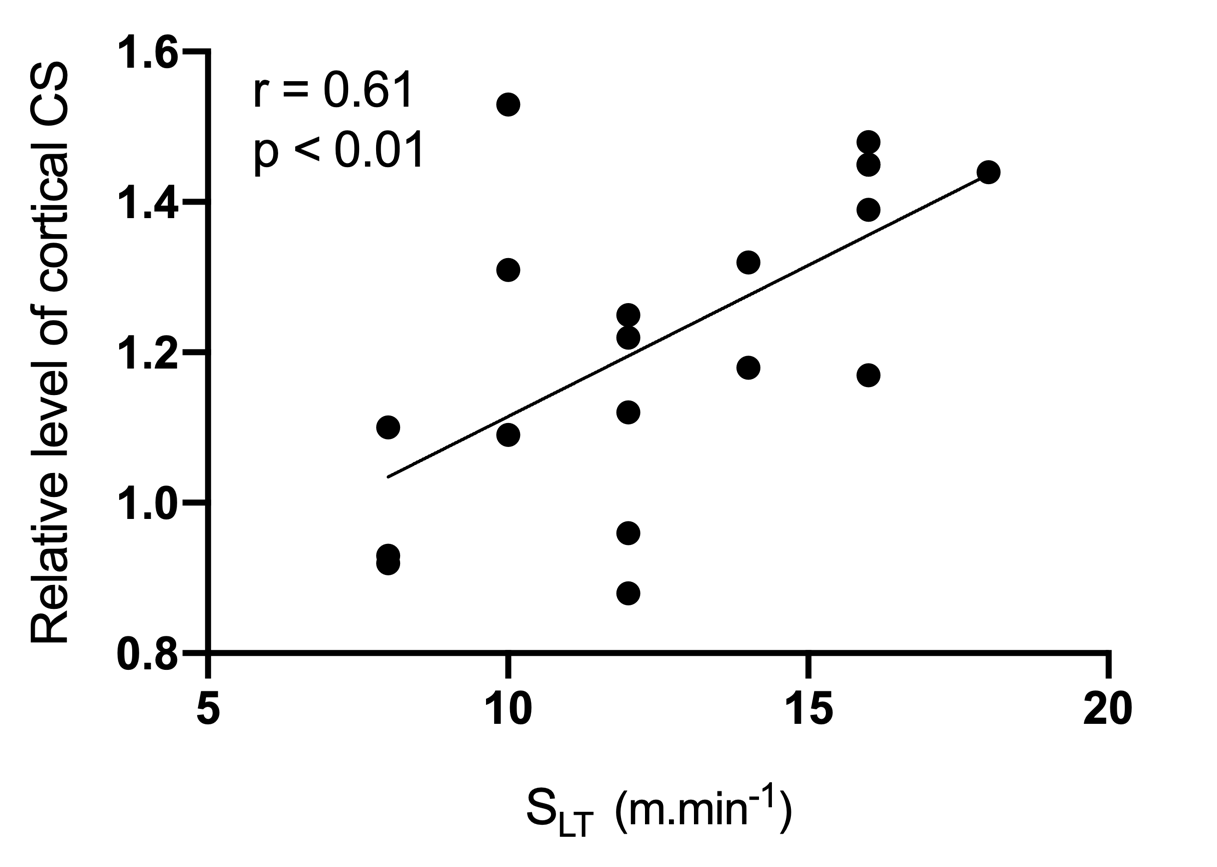 | D)  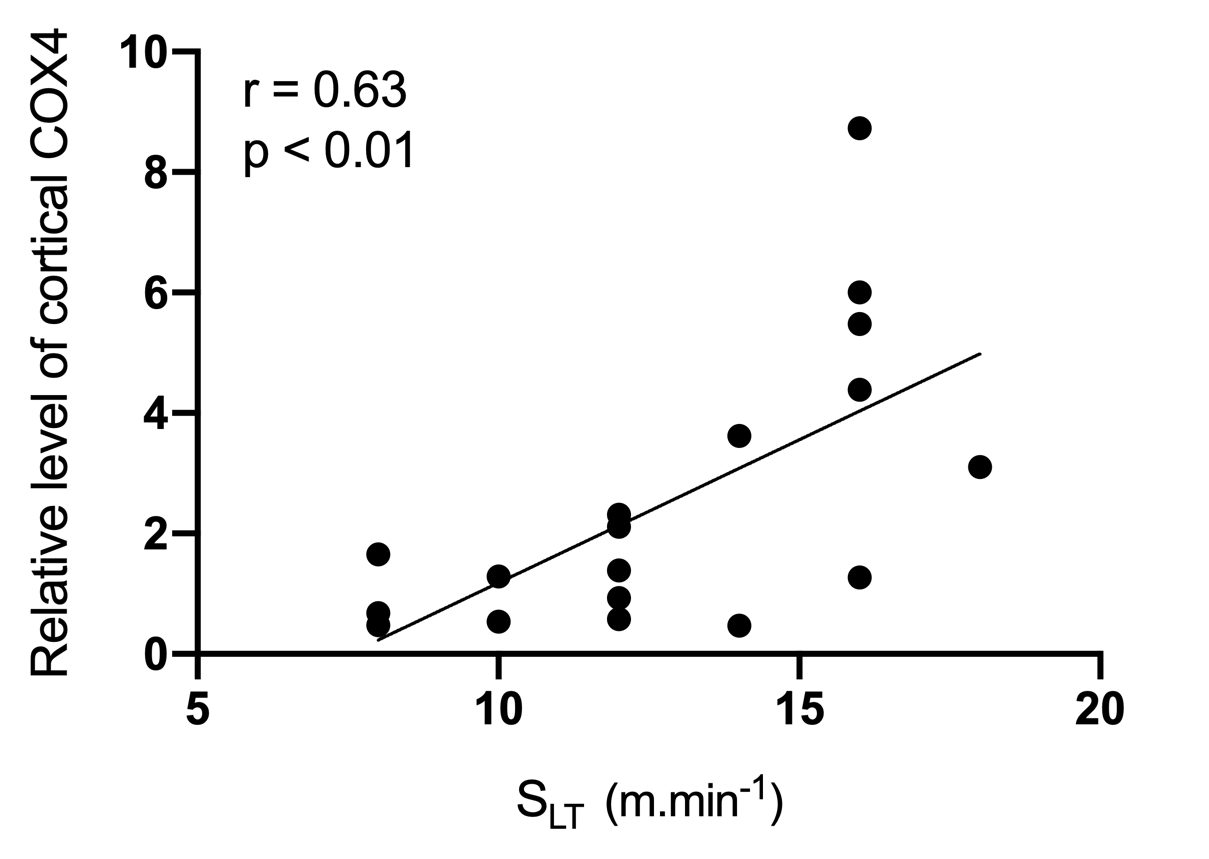 |
| E) 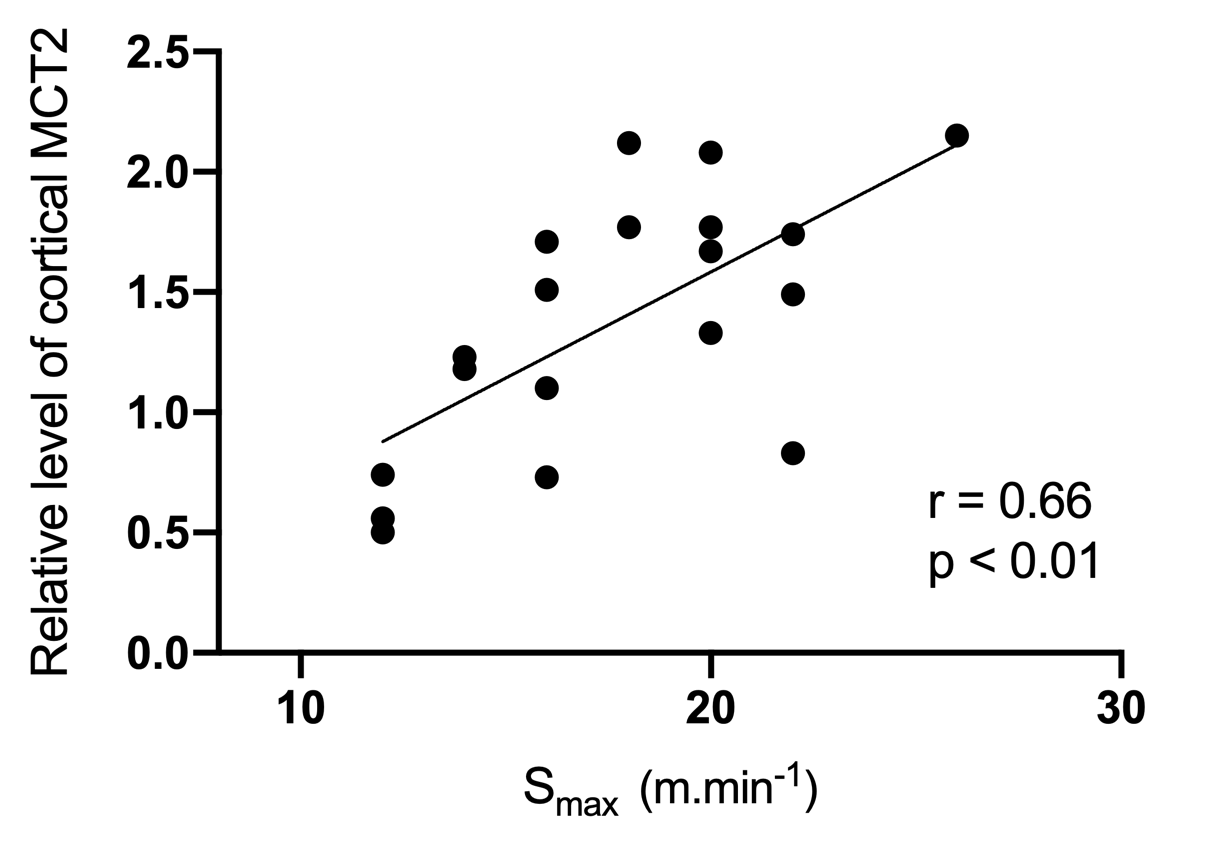 | F)  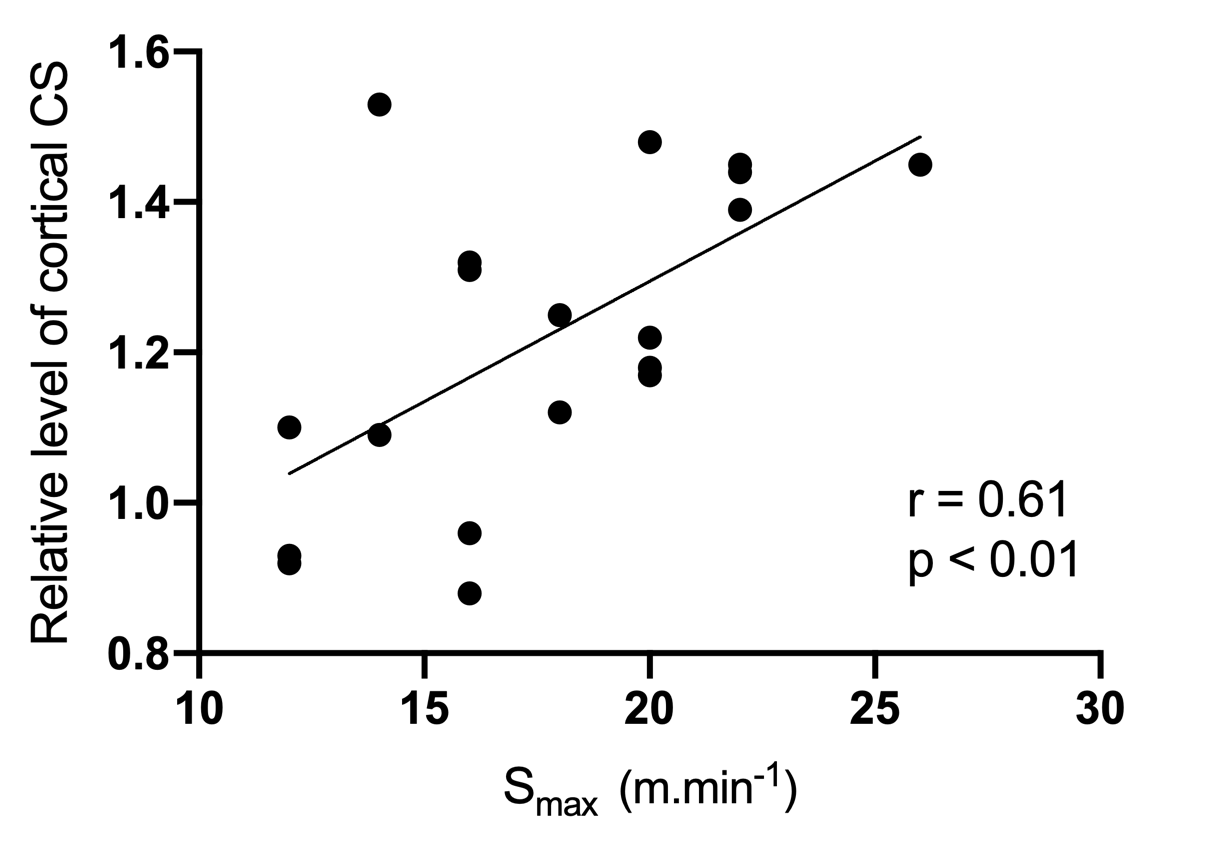 |
| G)  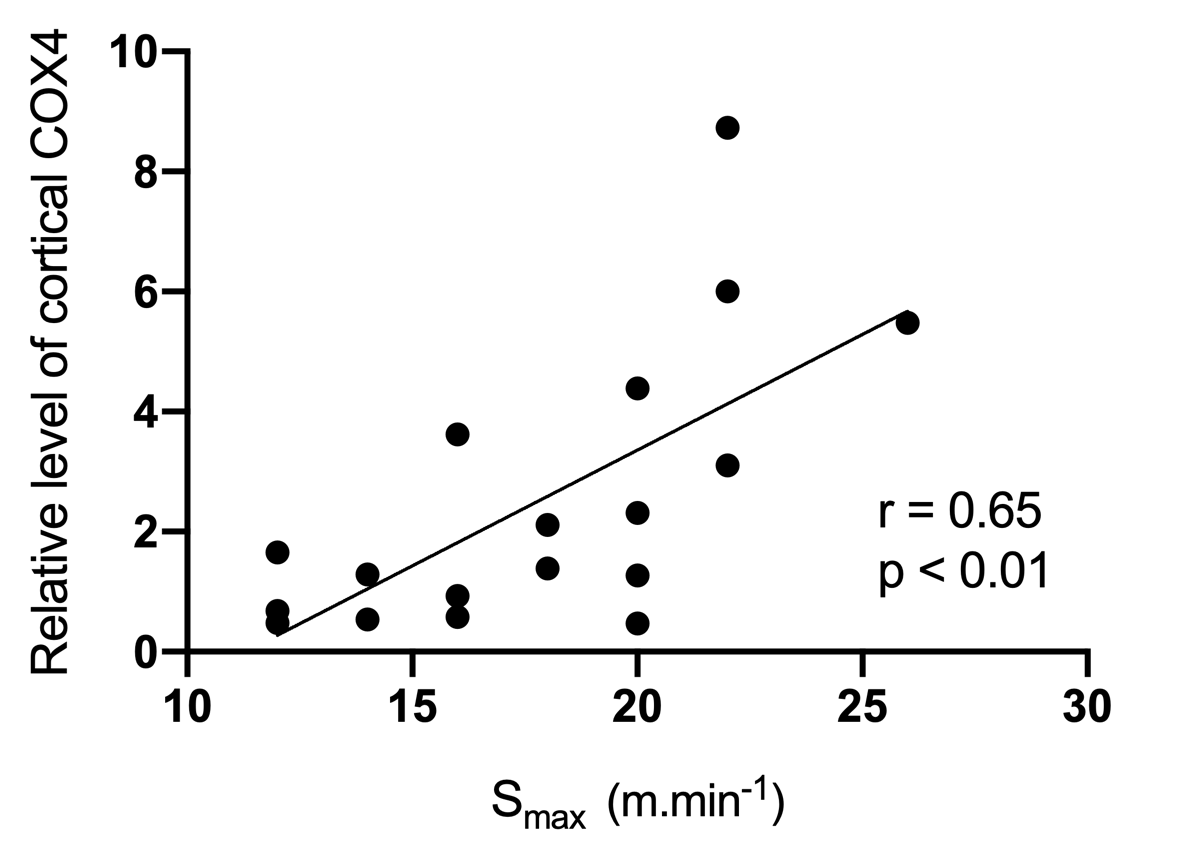 |  |
| **Figure S16: Correlation between energy metabolic markers in the cortex and S_LT_ and S_max_ after 4 weeks of training.** Pearson correlation between S_LT_ and VEGF (A), MCT2 (B), CS (C). Spearman correlation between S_LT_ and COX4 (D). Pearson correlation between S_max_ and MCT2 (E) and CS (F). Spearman correlation between S_max_ and COX4 (G). Pearson and Spearman correlation with two-tailed significance was conducted (n = 18-19, pooled samples of control, MICT, and HIIT groups). | |

| A)  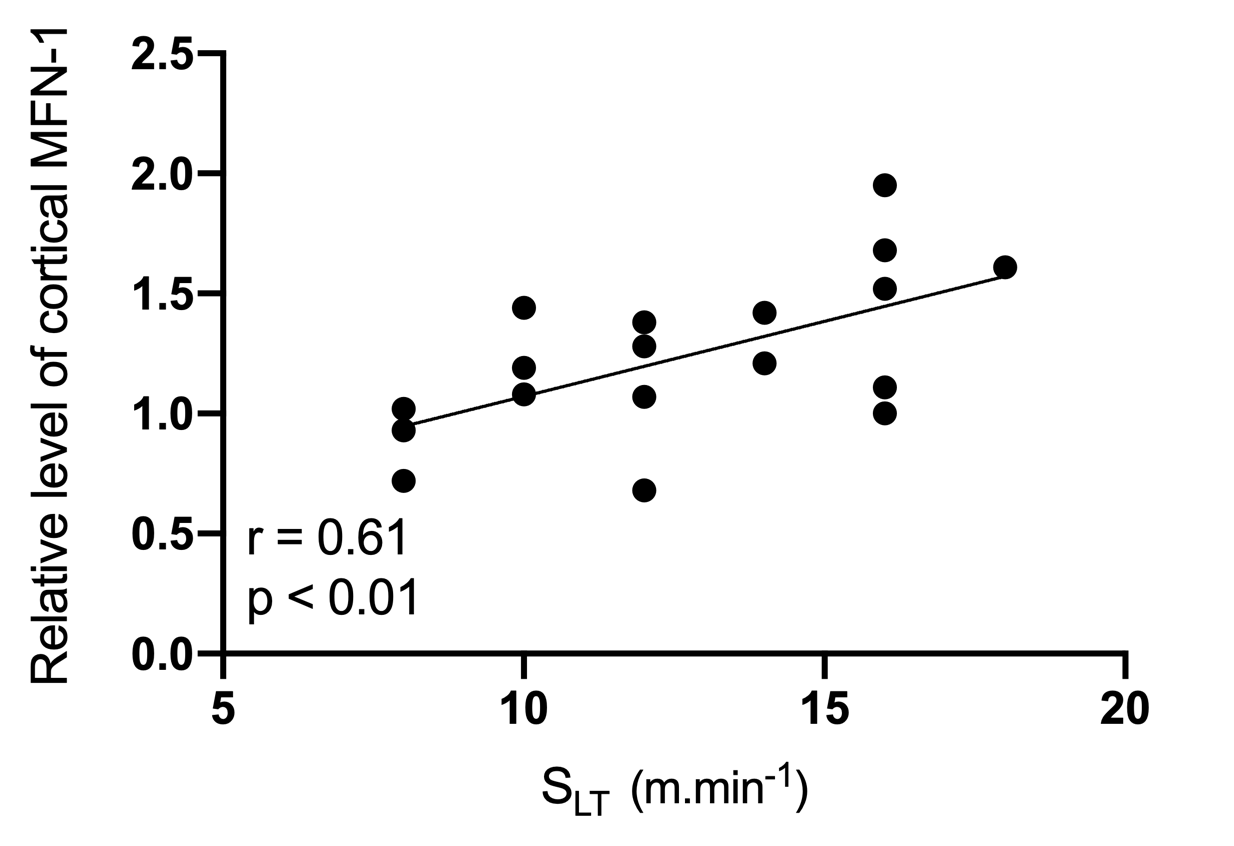 | B)  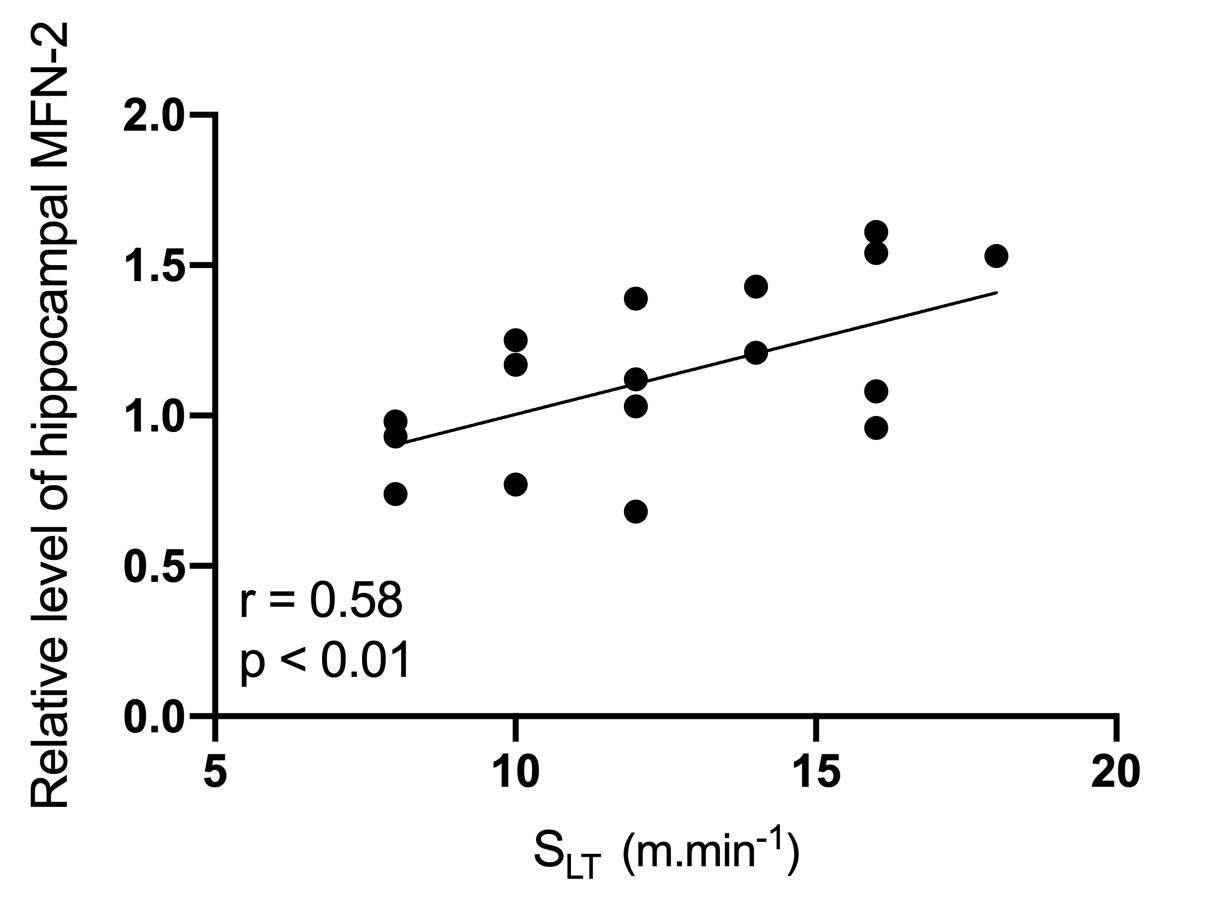 |
| --- | --- |
| C)  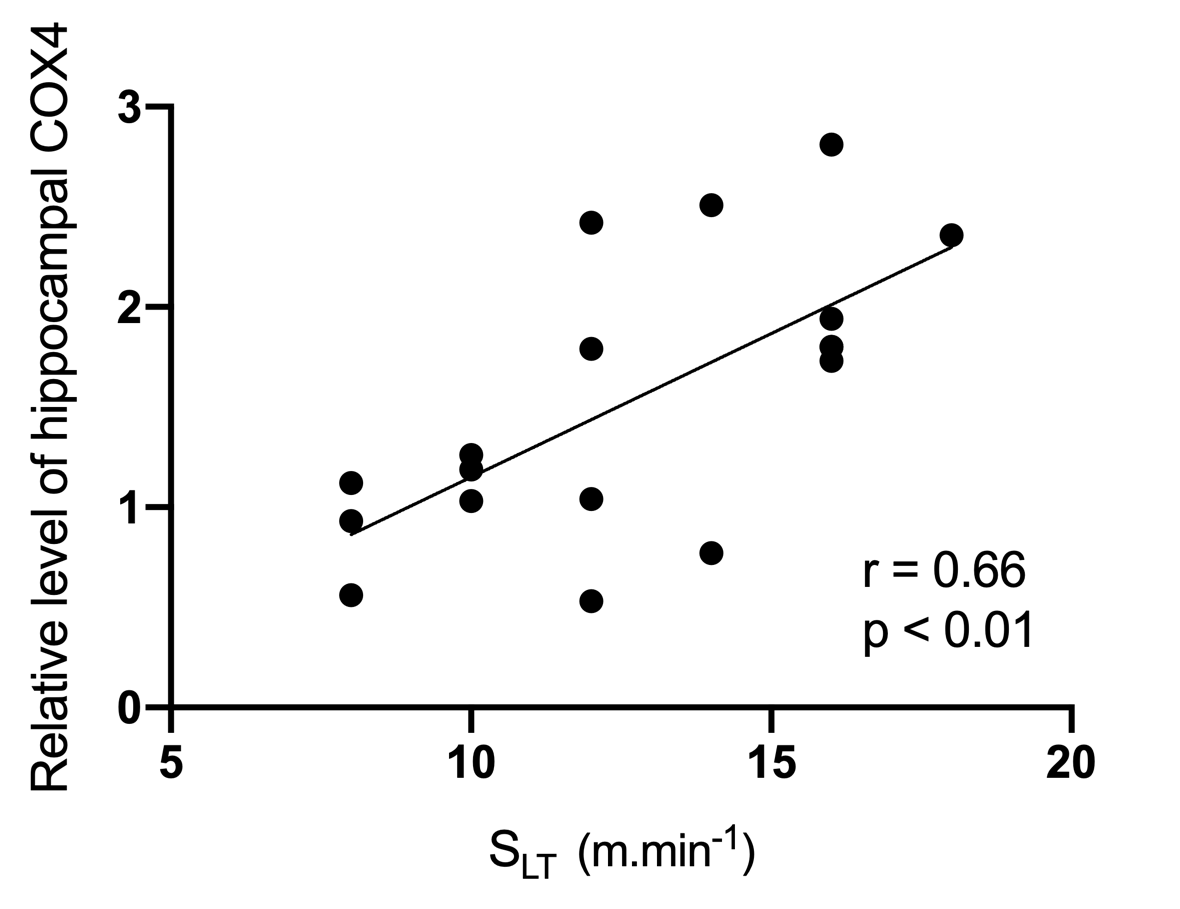 | D)  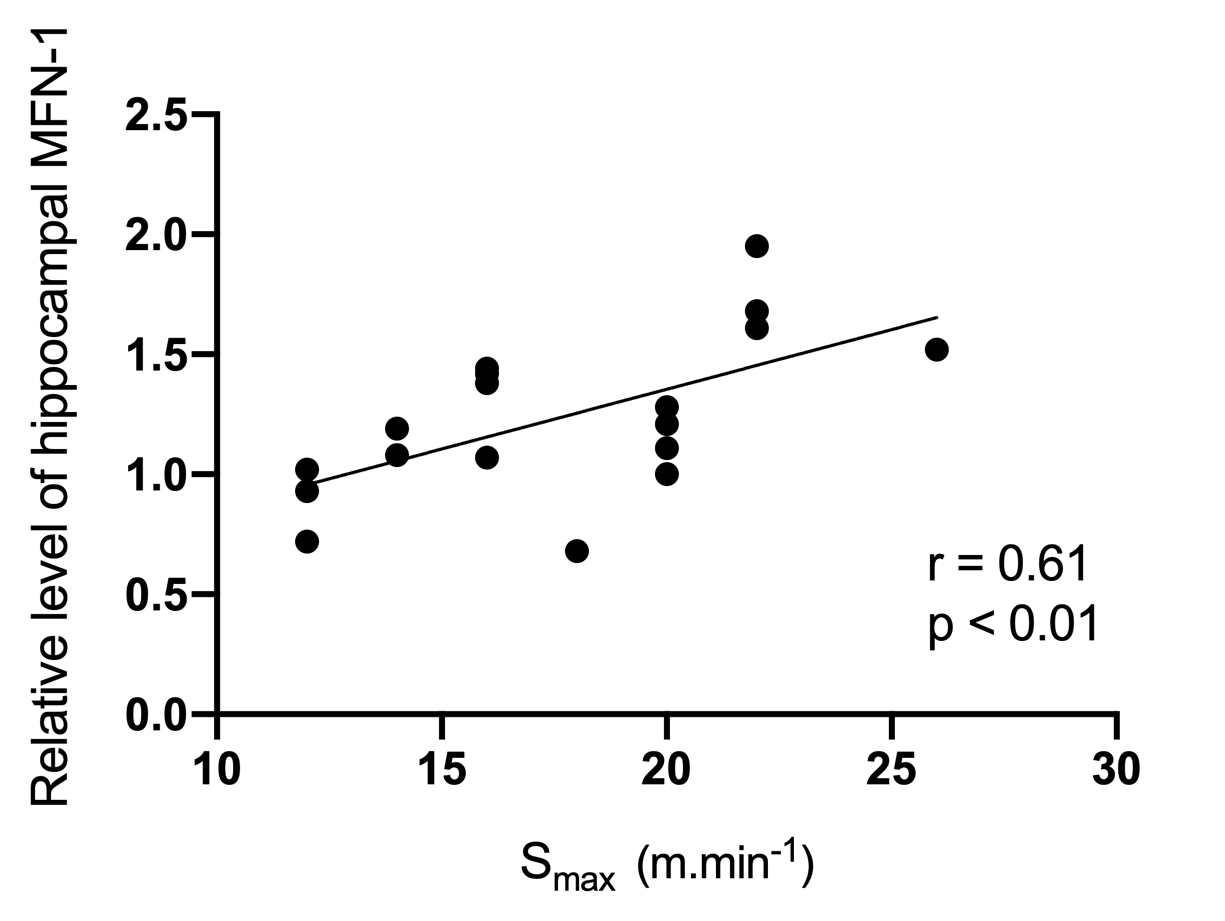 |
| E)  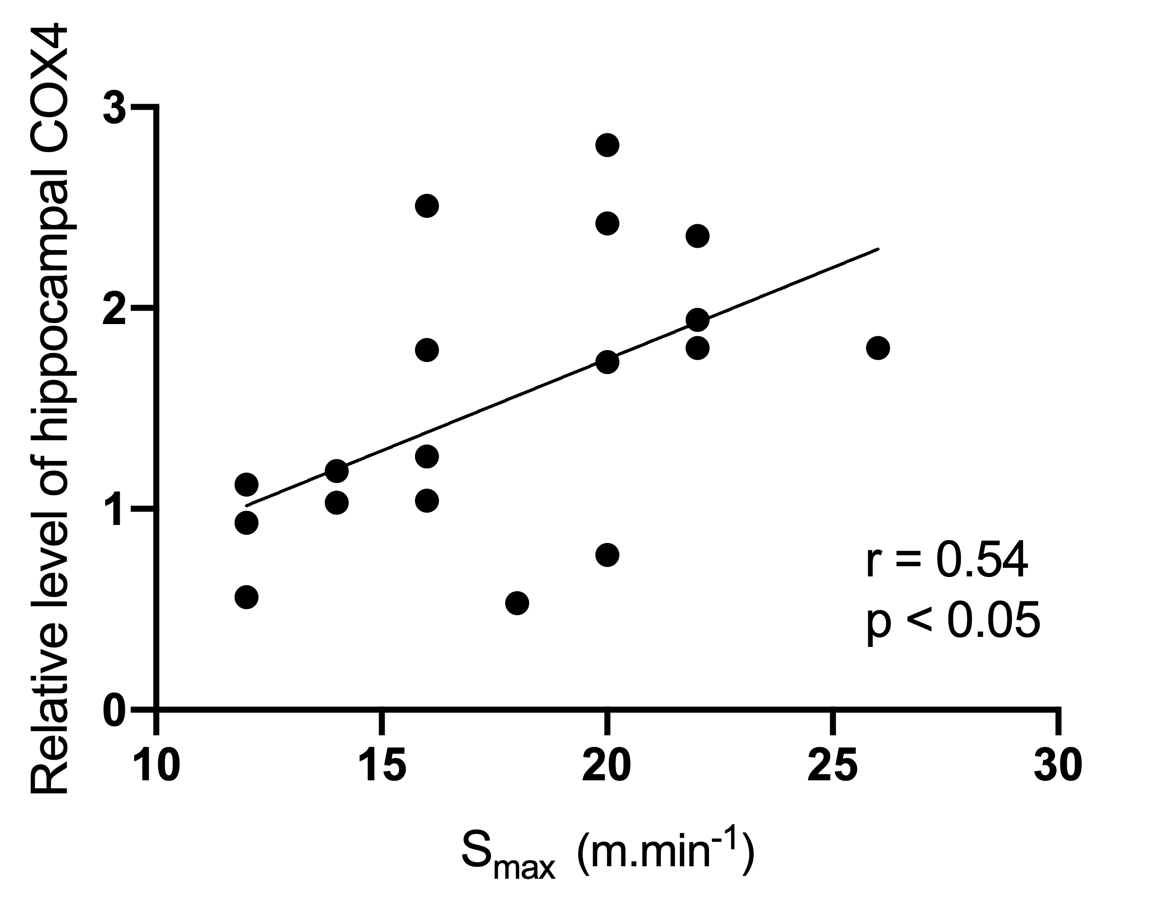 |  |
| **Figure S17: Correlation between energy metabolic markers in the hippocampus and S_LT_ and S_max_ after 4 weeks of training.** Pearson correlation between S_LT_ and MFN1 (A) and MFN2 (B). Spearman correlation between S_LT_ and COX4 (C). Pearson correlation between S_max_ and MFN1 (D). Spearman correlation between S_max_ and COX4 (E). Pearson and Spearman correlation with two-tailed significance was conducted (n = 18-19, pooled samples of control, MICT, and HIIT groups). | |
